# Controlling Excited State Localization in Bichromophoric Photosensitizers via the Bridging Group

**DOI:** 10.1021/acs.inorgchem.3c04110

**Published:** 2024-03-04

**Authors:** Georgina E. Shillito, Dan Preston, James D. Crowley, Pawel Wagner, Samuel J. Harris, Keith C. Gordon, Stephan Kupfer

**Affiliations:** † Institute of Physical Chemistry, Friedrich Schiller University Jena, Helmholtzweg 4, 07743 Jena, Germany; ‡ Research School of Chemistry, Australian National University, Canberra, ACT 2600, Australia; § Department of Chemistry, University of Otago, 362 Leith Street, Dunedin 9016, New Zealand; ∥ University of Wollongong, Northfields Avenue, Wollongong, NSW 2522, Australia; @ MacDiarmid Institute for Advanced Materials and Nanotechnology, Wellington, 6012, New Zealand

## Abstract

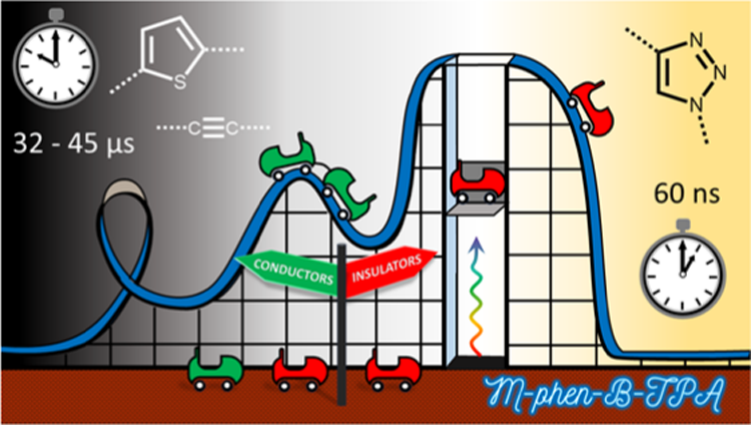

A series of photosensitizers comprised of both an inorganic and an organic chromophore are investigated in a joint synthetic, spectroscopic, and theoretical study. This bichromophoric design strategy provides a means by which to significantly increase the excited state lifetime by isolating the excited state away from the metal center following intersystem crossing. A variable bridging group is incorporated between the donor and acceptor units of the organic chromophore, and its influence on the excited state properties is explored. The Franck–Condon (FC) photophysics and subsequent excited state relaxation pathways are investigated with a suite of steady-state and time-resolved spectroscopic techniques in combination with scalar-relativistic quantum chemical calculations. It is demonstrated that the presence of an electronically conducting bridge that facilitates donor–acceptor communication is vital to generate long-lived (32 to 45 μs), charge-separated states with organic character. In contrast, when an insulating 1,2,3-triazole bridge is used, the excited state properties are dominated by the inorganic chromophore, with a notably shorter lifetime of 60 ns. This method of extending the lifetime of a molecular photosensitizer is, therefore, of interest for a range of molecular electronic devices and photophysical applications.

## Abstract 2

A series of bichromophoric transition metal photosensitizers are investigated in a joint synthetic, spectroscopic, and theoretical study. Selection of the bridging unit between the organic donor and acceptor dramatically influences both the excited state lifetime and its character.

## Introduction

In the design of photoactive systems, whether they are for photocatalytic,^1−4^ environmental sensing,^5−7^ photodynamic therapy,^8−11^ or photovoltaic applications,^12,13^ the light-harvesting molecule or moiety plays a crucial role in the overall functionality and efficiency of the system. The molecular design is influenced by the desired photophysical properties, be they achieving ligand loss such as in the case of photoCORMS^14,15^ and in some photocatalysts,^16,17^ unidirectional electron transfer to a specific acceptor moiety^18,19^ or the reduction of charge recombination rates^20^ and the creation of long-lived excited states.^21−25^ Whatever the desired photophysical behavior, a fundamental understanding of the photophysical and photochemical processes involved is vital to the photosensitizer’s design.

The design of photoactive transition-metal complexes has greatly expanded from a simple donor–acceptor structure, with numerous structural combinations frequently involving multiple donating and accepting groups present in the literature.^2,26−30^ Increasing the excited state lifetime of the photosensitizer is desirable in many applications, allowing greater time for additional processes and/or reaction steps to occur,^1,2,8^ e.g., subsequent electron or energy transfer events that may lead to the activation of a catalytic unit. One means by which this can be achieved is through the incorporation of large aromatic chromophores. This can facilitate a population of triplet ligand-centered (^3^LC) states, which typically have much longer lifetimes than their triplet metal-to-ligand charge-transfer (^3^MLCT) state counterparts due to their localization away from the metal center.^3,23,31−36^ For example, a recent study by Choroba et al. investigated pyrene-substituted Re(I) complexes with room-temperature phosphorescent lifetimes greater than 1000 μs.^23^ Similarly, a study by Wenger and co-workers showed that naphthalene was able to function as a triplet reservoir in an Ir(III) complex, leading to lifetimes of 72.1 μs.^3^ Excited state lifetimes may also be increased through the formation of intraligand charge-transfer (ILCT) states, which involve incorporation of an organic-based donor substituent.^37−41^

Many photoactive structures also incorporate a bridging group or groups between the donor and acceptor moieties, which can subsequently affect the excited state properties and electron and or energy transfer processes.^17,28,42−48^ For example, thiophene units are frequently found in polymers as they are well known for their conducting ability, improving π-conjugation and facilitating charge transfer.^49−54^ Thiophenes have also been successfully incorporated into donor–acceptor dyes and have been shown to red-shift as well as increase the intensity of the charge-transfer transition in both organic and inorganic systems.^42,45,55,56^ Ethynyl bridges have also been shown to increase donor–acceptor communication in a similar manner, providing a means of connecting different moieties in a rigid, planar geometry due to the sp-hybridized carbons.^38,57−60^ Robson et al. studied a family of Ru(II) terpyridine complexes with triphenylamine (TPA) donors connected through ethynyl and thiophene bridges.^49^ They found that the lowest energy state was ILCT in nature and was red-shifted by the inclusion of both ethynyl and thiophene groups. Contrastingly, 1,2,3-triazole containing bridges function as electronic insulators, significantly decreasing the oscillator strength of the transition.^42,57,60−62^

**Figure 1:**
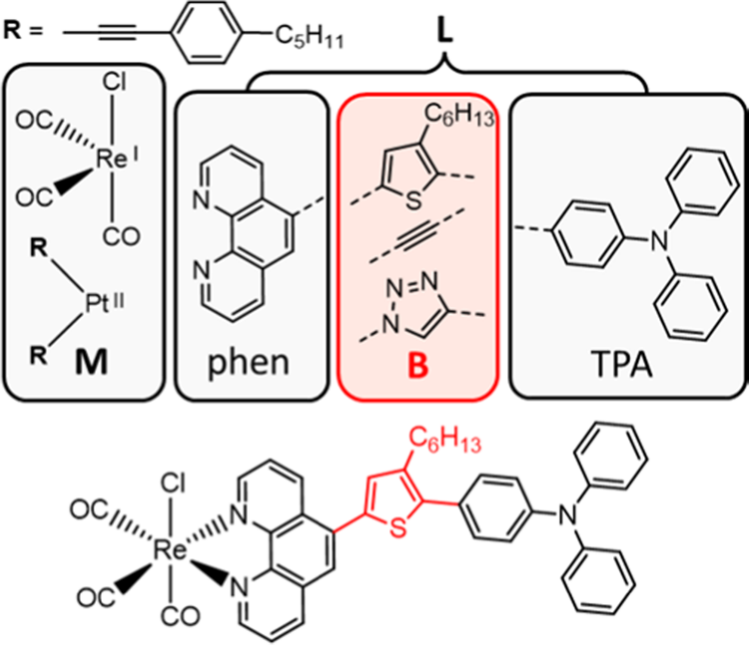
Structure of the complexes studied. The transition metal (M) corresponds to a ReCl(CO)_3_ or a Pt(R)_2_ core, and the ligand is comprised of a 1,10-phenanthroline acceptor (phen), a variable bridging unit (B, indicated in red), and a triphenylamine (TPA) donor group. The dashed lines indicate the connection positions. The complexes are subsequently labeled with the notation **M**–**L**, where **M** = Re or Pt, **L** = phen-thiophene-TPA (**thio-TPA**), phen-ethynyl-TPA (**CC-TPA**) and phen-1,2,3-triazole-TPA (**trz-TPA**). The structure of **Re-thio-TPA** is shown for reference.

Our recent work has examined the effect of a range of bridging groups on the electronic transitions in a series of Ru(II) and Re(I) complexes containing a dipyrido[3,2-*a*:2′,3′-*c*]phenazine (dppz) acceptor and a TPA donor.^38,42^ The bridge was shown to have a significant effect on the electronic properties of the ground state; however, the lowest energy excited state was consistently ^3^ILCT in character. Herein, the role that a bridging group plays on the photophysical properties of a new series of bichromophoric transition-metal complexes is investigated. The structures shown in Figure 1 contain a metal center and an organic ligand composed of a 1,10-phenanthroline (phen) acceptor, a variable bridging unit, and a TPA donor group. Previous investigations have demonstrated that increasing the energy of the acceptor by changing it from a dppz to a phen moiety can have a significant impact on the excited state properties in Re(I) and Pt(II) phen-TPA systems without a bridging unit.^39,63^ We therefore endeavor to explore the role of a bridging group in these already photophysically rich systems by applying both steady-state and time-resolved spectroscopic methods in combination with scalar-relativistic quantum chemical calculations. Good communication between donor and acceptor moieties is desirable in many applications, so the role of a conducting thiophene bridge is initially investigated, exploring its effect on the FC photophysics as well as the excited state behavior. The properties of complexes containing an ethynyl bridging group, which has similar electronic properties while avoiding geometric factors such as bridge rotation, are also explored. Finally, in some cases, the ability to isolate excited states or disrupt specific electron transfer pathways may also be of interest; therefore, as a point of comparison, the effect of incorporating the electronically insulating 1,2,3-triazole bridge was also investigated.

## Experimental Section

### Synthesis

The rhenium(I) complexes were synthesized through the 1:1 combination of [Re(CO)_5_Cl] and the appropriate ligand in toluene with heating at 80 °C overnight. They were isolated through either the removal of the solvent or precipitation. The platinum(II) complexes were synthesized through the 1:1 combination of [Pt(COD)(4-pentylphenylacetylide)_2_]^63^ in 1:5 CH_3_CN/CH_2_Cl_2_ with heating at 45 °C for 3 days under a nitrogen atmosphere. They were purified by column chromatography on silica. Full details can be found in the Supporting Information.

### Spectroscopy

Electronic absorption and resonance Raman spectroscopy, utilizing excitation wavelengths across the lowest energy absorption band, were used to investigate the FC photophysics. The excited state properties were studied using a combination of nanosecond (ns) transient absorption (TA) spectroscopy with 354.7 nm excitation, photon-counting measurements, and ns time-resolved resonance Raman (TR^3^) (λ_pump_ = 354.7 nm, λ_probe_ = 354.7 nm). Further experimental details can be found in the Supporting Information.

### Computational Details

All quantum chemical calculations were performed using the Gaussian 16 program^64^ (B.01) (scalar-relativistic simulations were performed using Orca^65,66^—see details in the Supporting Information). The singlet ground-state geometries of the investigated structures were obtained at the density functional theory (DFT) level. A B3LYP^67,68^-based functional denoted B3LYP35^39,69−73^ comprising 35% exact-exchange and 58.5% of non-local B88^74^ exchange, and the LYP^68^ correlation was employed for the complexes, while the CAM-B3LYP^75^ functional was used for the organic ligand structures. The split-valence def2-SVP basis set^76^ was used unless stated otherwise, alongside Grimme’s D3 dispersion correction^77^ with Beck–Johnson damping to account for long-range interactions. An implicit CH_2_Cl_2_ solvent field was incorporated with the integral equation formalism, employing the SMD model.^78^ Time-dependent DFT (TDDFT) calculations were performed by using the same respective functionals. Further details regarding the rotamers, basis set effects on excited state properties (def2-SVP vs def2-TZVP), and simulated spectra can be found in the Supporting Information. The optimized S_0_ and T_1_ geometries of the structures, including the various rotamers, are available in ref (79) via the open data repository Zenodo.

## Results and Discussion

### Franck–Condon Photophysics (**M-thio-TPA**)

The ground-state structures of the ligand and both thiophene complexes were modeled in solution by means of density functional theory (DFT) incorporating an implicit solvent field.^78^ However, due to the flexible nature of the thiophene bridge, different ligand orientations are possible in solution. A relaxed scan was therefore performed, whereby the dihedral angle between the phen and thiophene groups was altered in a stepwise manner. As shown in Table S1, four possible rotamers were identified in the ligand and both thiophene complexes, with energies that vary by only 0.02 eV for the ligand and 0.03 eV for the complexes. These structures were confirmed to be energetic minima via frequency calculations.^80^

**Figure 2:**
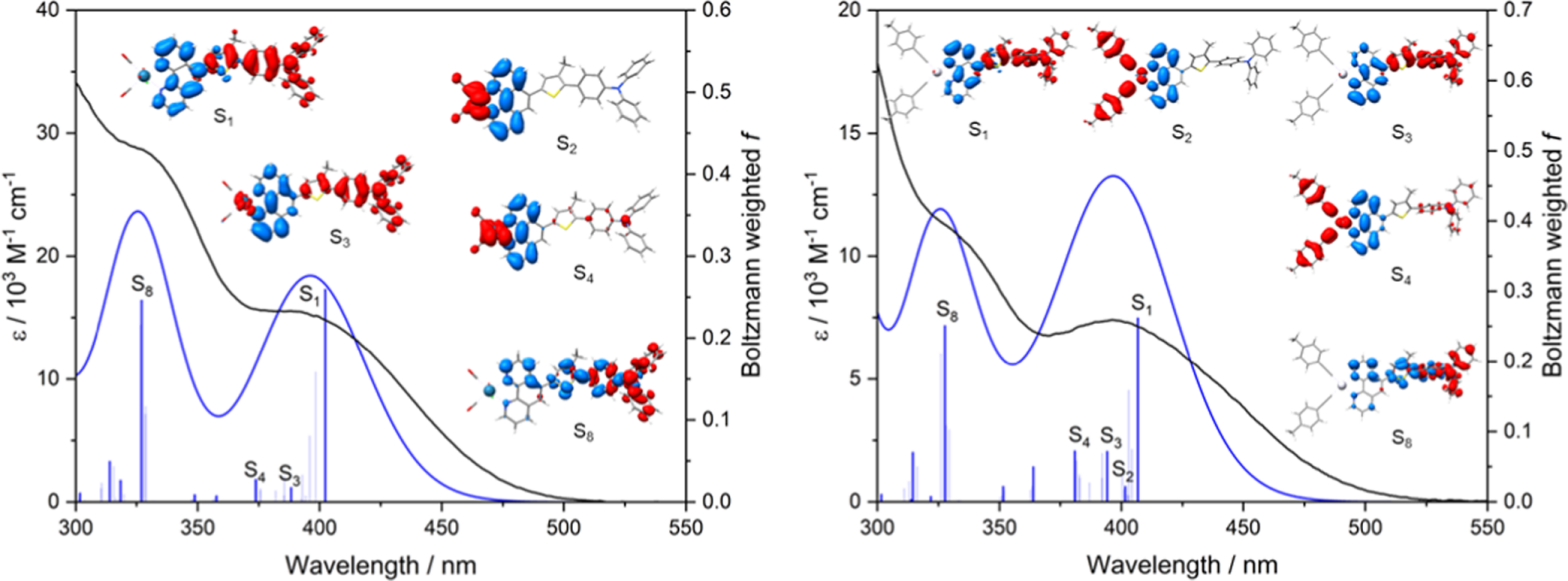
Experimental (in black) and simulated (in blue; B3LYP35, def2-SVP, CH_2_Cl_2_ solvent field) electronic absorption spectra of **Re-thio-TPA** (left) and **Pt-thio-TPA** (right) in CH_2_Cl_2_. The simulated spectra were obtained via a Boltzmann weighted distribution of the four identified rotamers, with the bold blue vertical lines corresponding to the transition energies for the lowest energy rotamer (rotamer A). The charge-density differences (CDDs) are shown for key transitions for rotamer A, with the electron density moving from red to blue.

The simulated electronic absorption spectra, obtained at the TDDFT level of theory, and the oscillator strengths shown in Figure 2 (and Figure S1) were determined via a Boltzmann weighted distribution of the four rotamers, with the transitions shown in bold attributed to the lowest energy rotamer. The higher energy shoulder in the electronic absorption spectrum at approximately 330 nm is attributed to the S_8_ electronic state, which possesses a mixture of ILCT and LC character. The calculations predict that the lowest energy absorption band is primarily attributed to an ILCT transition (S_1_) from the TPA donor to the phen acceptor at just over 400 nm for both the Re(I) and Pt(II) complexes (Figure S2). This is well matched with the experimentally observed λ_max_ at 397 and 406 nm, respectively. Additional weaker transitions of ILCT, MLCT, and mixed character (S_2_–S_4_) are also predicted to occur in this region. As shown in the charge-density difference (CDD) insets, the ILCT states (S_1_ and S_3_) of the Re(I) and Pt(II) species both involve significant electronic contributions from the thiophene bridge as well as the TPA donor. Notably, the nature of the phen-localized acceptor orbital differs between the two ILCT states, while the donor orbital remains the same, consistent with previous studies.^39,63^ Interestingly, unlike in **Pt-thio-TPA**, the S_3_ state in **Re-thio-TPA** is not purely ILCT in nature, as it contains some mixed contribution from the metal. The respective energies, oscillator strengths, and order of the states are slightly affected by the nature of the rotamer (see Tables S2–S11); however, the lowest energy absorption band is primarily attributed to a strongly dipole-allowed ILCT transition.

TDDFT calculations performed along this dihedral angle (Figures S3 and S4) reveal that the energy of the S_1_ ILCT state fluctuates in a similar manner to the S_0_ energy, reaching minima when the dihedral angle between the phen and the thiophene bridge is close to planar, increasing the electron delocalization throughout the ligand. In contrast, the MLCT energy is stabilized as the planarity is decreased. The S_3_ ILCT state is consistently higher in energy and lower in oscillator strength than S_1_ within the energetic ground-state minima; however, the relative intensities and energies of these two states are slightly modulated by the dihedral angle. For example, the two lowest energy rotamers of **Re-thio-TPA** (rotamers A and B) with dihedral angles of ±45° show the greatest and lowest S_1_ and S_3_ intensities, respectively, while in the slightly less planar rotamers C and D, with dihedral angles of ±125°, the opposite is true. In the case of rotamer D, the lowest ILCT state also gains some metal character and is raised in energy to become the S_2_ state. The orbital parentage of these states is discussed in further detail in the following section, as well as how resonance Raman spectroscopy was used to further support their characterization.

**Figure 3:**
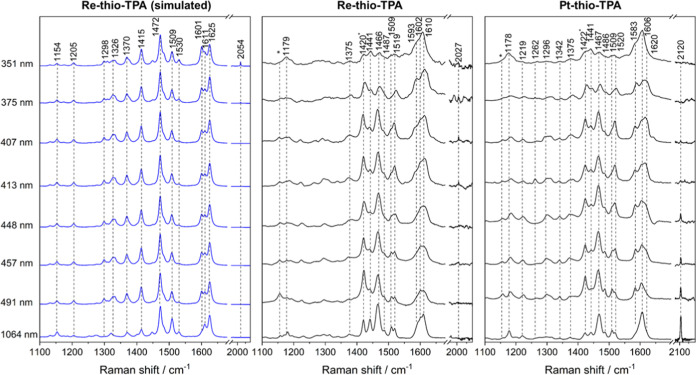
Simulated (left; B3LYP35, def2-SVP, CH_2_Cl_2_ solvent field) and experimental resonance Raman spectra of **Re-thio-TPA** (center) and **Pt-thio-TPA** (right) obtained at a range of excitation wavelengths, indicated on the left-hand side. A nonresonant 1064 nm FT-Raman spectrum obtained in a KBr disk is included for comparison. The simulated spectra were obtained from a Boltzmann weighted distribution of rotamers’ spectra; frequencies are scaled by 0.95 (left); and experimental resonance Raman spectra were obtained in CH_2_Cl_2_ (solvent bands indicated with an *).

Resonance Raman spectra of **Re-thio-TPA** shown in Figure 3 were obtained in a CH_2_Cl_2_ solution at multiple excitation wavelengths encompassing the lowest energy absorption band. Nonresonant 1064 nm FT-Raman spectra of solid samples in KBr disks were also measured for comparison. The edge of the 330 nm shoulder is probed with 351 nm excitation, and predominantly TPA-based vibrations^39,81−84^ at 1606 and 1178 cm^–1^ are enhanced, consistent with a TPA-localized LC state. As the excitation wavelength is tuned to red, a number of other ligand-based vibrations grow in intensity. Delocalized vibrations at 1375 and 1441 cm^–1^, which involve the whole ligand, are enhanced as well as phen localized modes at 1422 (coincident with a CH_2_Cl_2_ mode), 1520, and 1584 cm^–1^.^85,86^ The strong band at 1467 cm^–1^ has a significant thiophene character and shows strong enhancement with excitation wavelengths between 407 and 491 nm.^87^ The enhancement pattern is consistent with the formation of an ILCT state and highlights the active role that the bridge plays in the low-energy electronic states, consistent with the TDDFT predictions.

While the spectra of the Re(I) and Pt(II) species are very similar, the 1583 cm^–1^ phen mode is more strongly enhanced at longer excitation wavelengths in **Pt-thio-TPA**. These slight differences in the resonance enhancement pattern are successfully replicated in the simulated spectra, shown in blue in Figures 3 and S5, for **Re-thio-TPA** and **Pt-thio-TPA**, respectively. Examination of the molecular orbitals involved in the low energy, strongly absorbing ILCT transition reveals primary contributions from a TPA-localized highest occupied molecular orbital (HOMO, orbital numbers 173 and 206 for **Re-thio-TPA** and **Pt-thio-TPA**, respectively) to two separate π_phen_^*^ orbitals, corresponding to the LUMO (lowest unoccupied molecular orbital) and LUMO + 1. These unoccupied orbitals have pseudo b_1_ and a_2_ symmetry, respectively, based on the *C*_2_*_v_* description of the isolated phen component of the ligand,^88^ with the electron density of the b_1_ acceptor orbital (orbital numbers 174(**Re-thio-TPA**) and 207(**Pt-thio-TPA**)) localized toward the center of the coordination sphere, while the electron density distribution of the a_2_ acceptor orbital (orbital numbers 175(**Re-thio-TPA**) and 208(**Pt-thio-TPA**)) is more localized at the periphery of the coordination sphere, i.e., in closer proximity to the bridging moiety. Therefore, as shown in Table S12, the S_1_ state of **Re-thio-TPA** can be described by weighted contributions of electron transfer between the orbitals 173 → 174(b_1_) and 173 → 175(a_2_), of 34 and 51%, respectively. Similarly, the S_1_ state of **Pt-thio-TPA** can therefore be described as a transition between 206 → 207(b_1_) and 206 → 208(a_2_) with respective weightings of 49 and 33%. Therefore, the π(b_1_)_phen_^*^ orbital has a greater contribution to the S_1_ ILCT transition in **Pt-thio-TPA** than in **Re-thio-TPA**. The greater involvement of the b_1_ orbital (with its greater electron density toward the center of the coordination sphere) in the Pt(II) complex is likely responsible for the increased resonance enhancement of the 1583 cm^–1^ mode, which, as depicted in Figure S5, shows vibrational motion more closely associated with this orbital distribution. In addition, in both complexes, the aforementioned higher-lying S_3_ ILCT state shows the same orbital contributions but with the opposing dominant acceptor orbital to that involved in the S_1_ state. Thus, we observed two ILCT transitions with differently weighted orbital parentage, as seen in previous studies.^39^ Lastly, it should be noted that the a_2_ acceptor orbital exhibits double bond character with respect to the bridge and will, therefore, be heavily involved in the excited state relaxation.

### Excited State Photophysics (**M-thio-TPA**)

**Figure 4:**
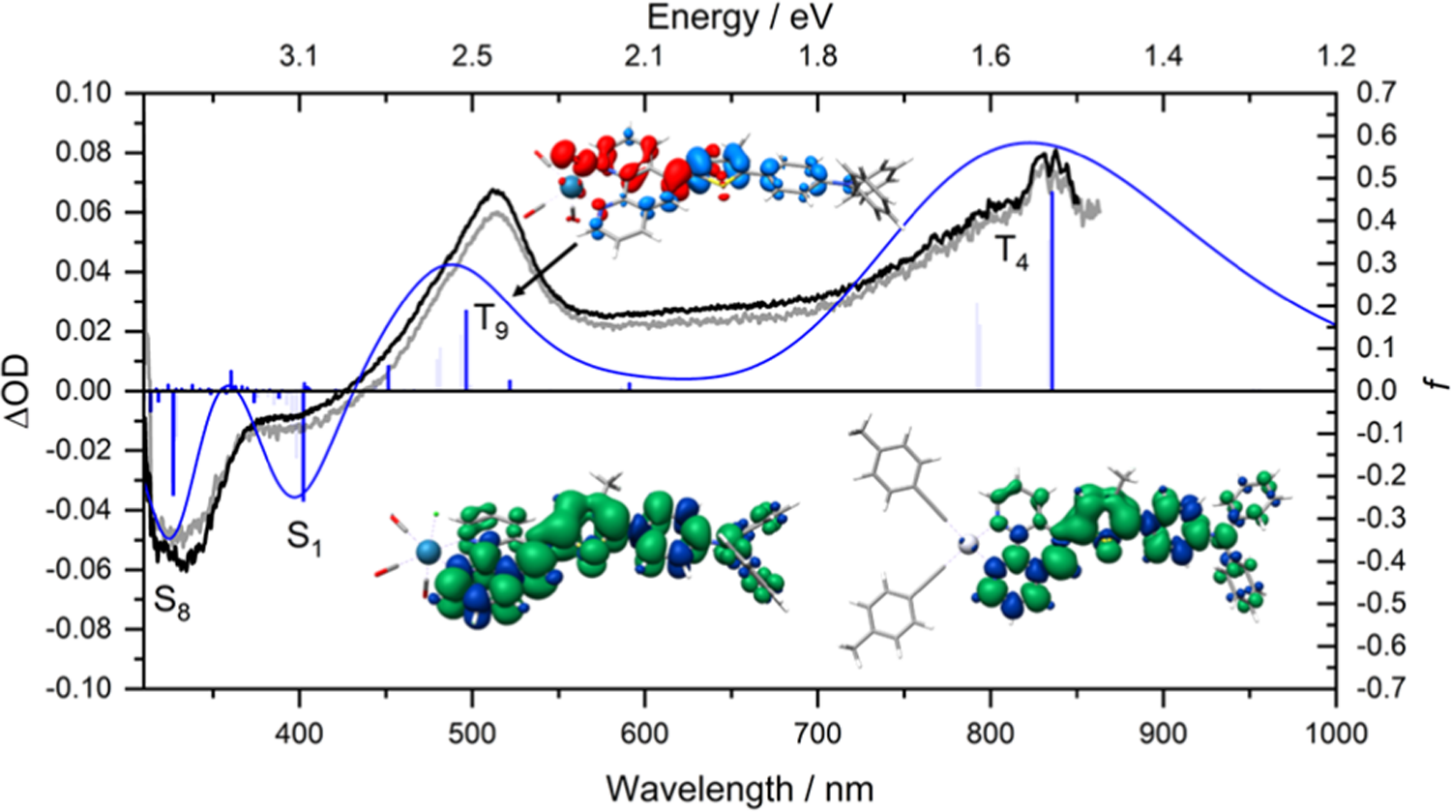
Boltzmann weighted simulated (blue; B3LYP35, def2-SVP, CH_2_Cl_2_ solvent field) and experimental (black) transient absorption spectra of **Re-thio-TPA** obtained with 354.7 nm pulse excitation in degassed CH_2_Cl_2_. The downward vertical bars correspond to ground-state bleach (singlet–singlet transitions in the S_0_ equilibrium), while upward vertical bars are associated with excited state absorption (triplet–triplet transitions in the T_1_ structure). Bold bars correspond to rotamer A. The oscillator strengths have been scaled based on the Boltzmann weighting. The top inset shows the CDD for the T_9_ state. The lower inset shows the spin density distribution of **Re-thio-TPA** (left) and **Pt-thio-TPA** (right) in their optimized T_1_ geometry, which would likely be obtained from excitation of the S_0_ rotamer A. The experimental spectrum of **Pt-thio-TPA** is also shown in faded gray for comparison.

The triplet ground states of the thiophene complexes were also optimized, and as per the singlet structures, four rotamers were identified (Table S1). The spin density distribution of each rotamer in both the Re(I) and Pt(II) species was consistently shown to be ^3^ILCT/^3^LC in nature, delocalized over the entire ligand system, as shown in Figure 4. The fully delocalized nature of the T_1_ spin density supports the near-planar ligand geometries obtained, as the planarity facilitates improved conjugation and electron delocalization over the ligand system, thereby minimizing the ^3^ILCT/^3^LC energy. Population of the previously discussed π(a_2_)_phen_^*^ acceptor orbital with the double bond character to the bridge leads to the planarization of this highly delocalized triplet state. This is in contrast to the primary dipole-allowed MLCT states, which involve the π(b_1_)_phen_^*^ orbital and hence do not promote ligand planarization.^89^

The excited state properties of the thiophene-containing complexes were investigated with nanosecond transient absorption spectroscopy and time-resolved resonance Raman, supported by TDDFT calculations. The complexes exhibited very weak and short-lived emission that could not be reliably measured with the 10 Hz, nanosecond pulse laser. The ns transient absorption spectra of the two complexes, obtained with 354.7 nm excitation, are given in Figure 4 and are almost identical, with the spectra of **Re-thio-TPA** and **Pt-thio-TPA** shown in black and gray, respectively. A strong ground-state bleach is observed at 330 nm, which extends out to 430 nm, after which the transient signal remains positive. A clearly resolved, strong signal is observed at 514 nm, with an additional prominent positive feature present at around 800 nm. These features are likely attributed to the thiophene bridge and the TPA^•+^, respectively.^90−93^ The simulated transient absorption spectra are an excellent match to the experimental results, with transitions from the T_1_ ground state to the T_4_ and T_9_ states responsible for the 514 and 800 nm absorption signals, respectively (Figures 4 and S6). The excellent replication of the experimental TA spectra suggests that the ^3^ILCT/^3^LC nature and geometry of the triplet state are well modeled. As illustrated in Figure S7, the transient absorption features do not evolve over time and simply decay to the ground state, with no new bands observed.

As shown in Table 1, kinetic traces obtained at 515 nm show long-lived biexponential decay, with lifetime components of 12.0 and 42.5 μs for **Re-thio-TPA**, and 14.8 and 45.3 μs for **Pt-thio-TPA**, with relative intensities of the τ_1_ and τ_2_ components of approximately 1:1 and 1:2 for the two thiophene complexes, respectively. The existence of biexponential decay means that the internal conversion rate between the two states must be slower than their respective nonradiative decay rates to the ground state and that both states have similarly weighted contributions. The long-lived nature of the excited states is attributed to significant ^3^LC character and decoupling from the metal center. This is consistent with the previously discussed spin density of the optimized triplet geometry and the fact that the two lowest triplet states have a mixture of ^3^LC and ^3^ILCT characters. Furthermore, photon-counting measurements of the ligand also showed biexponential decay, with 1.4 and 4.7 ns components (Figure S8); however, the decay is primarily attributed to the short-lived component, so the possibility that the longer-lived component is due to an impurity cannot be excluded.

**Table 1:** Transient Absorption Kinetics Data Obtained for the Complexes in Degassed CH_2_Cl_2_ Solutions with 354.7 nm Excitation^a^

	λ (nm)	τ_1_ (μs)	τ_2_ (μs)
**Re-thio-TPA**	515	12.0 (1)	42.5 (1)
**Pt-thio-TPA**	515	14.8 (1)	45.3 (2)
**Re-CC-TPA**	476	12.4 (1)	43.6 (3)
**Pt-CC-TPA**	476	11.2 (1)	31.7 (2)
**Re-trz-TPA**	520	0.06	
**thio-TPA**^b^	524	0.0012 (38)	0.0047 (1)
**trz-TPA**^b^	545	0.0139	

a λ indicates where the kinetic trace was measured. The approximate relative intensities of the biexponential components are given in brackets.

b Obtained through photon-counting measurements.

In line with our previous investigations, scalar-relativistic (SR) calculations performed utilizing the zero-order regular approximation (ZORA) and the Douglas–Kroll–Hess (DKH) approach^94,95^ in the FC geometry (Tables S13–S20) revealed strong spin–orbit coupling (SOC) between the initially populated ^1^MLCT states and upper ^3^MLCT states. This provides an “MLCT gateway” to allow the low-lying ^3^ILCT/^3^LC states (T_1_, T_2_) to be subsequently populated via internal conversion. These ligand-localized (triplet) states exhibit low SOC values with low-lying excited singlet states and, thus, are likely inaccessible directly via intersystem crossing. Given the prominent existence of rotamers in the investigation, it is important to consider the possibility that the biexponential nature of the decay could also be attributed to the decay from different rotameric structures present in solution. SR-TDDFT calculations were, therefore, also performed on all ground-state rotamers. However, no notable differences were observed, with excited states of near-equivalent energy, character, and SOC values obtained (Tables S13–S20). Therefore, it is unlikely that the presence of rotamers in solution is responsible for the biexponential decay; however, additional computational and experimental measurements were undertaken to examine this possibility and are briefly discussed in the final subsections.

**Figure 5:**
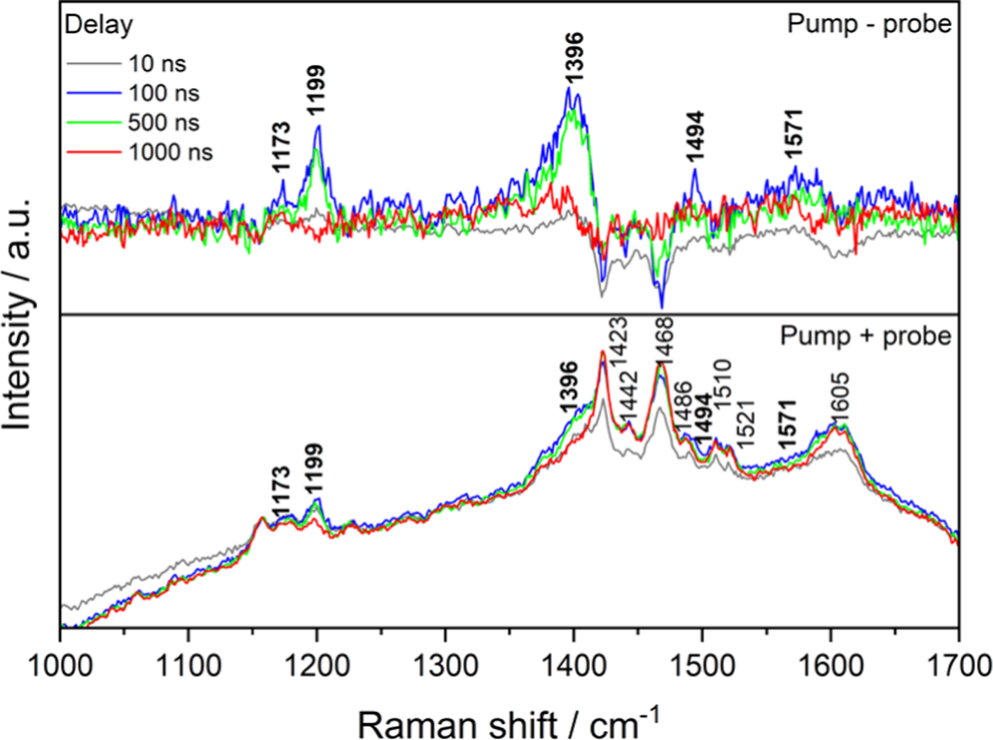
TR^3^ spectra of **Re-thio-TPA** obtained with different pump (λ_pump_ = 354.7 nm) and probe (λ_probe_ = 532.0 nm) delays in a degassed CH_2_Cl_2_ solution. The lower panel shows the pump + probe spectra normalized to the 1157 cm^–1^ solvent band, while the upper panel shows the result of subtraction of the “probe-only” spectrum. Excited state bands are indicated in bold.

The TA profile, combined with the long-lived excited states of **Re-thio-TPA** and **Pt-thio-TPA**, make them suitable complexes for investigation using ns time-resolved resonance Raman (TR^3^) spectroscopy. 354.7 nm pump and 532.0 nm probe pulses utilized to coincide with the ground-state bleach and excited state absorption, respectively, allowed a resonance Raman signal of the excited state to be obtained. The TR^3^ spectra of **Re-thio-TPA** are given in Figure 5 at multiple delays between the pump and probe pulses, where the 532.0 nm probe-only spectrum corresponds to a nonresonant spectrum of the ground state (Figure S9). The upper panel gives the result of subtracting the solvent-normalized probe-only spectrum to show the excited state signature, such that the growth of excited state features at 1173, 1199, 1396, 1494, and 1571 cm^–1^ can be observed, which grow and decay as a collective group. As shown in Figure 4, a probe wavelength of 532.0 nm is suitable to probe the strong excited state absorption at 515 nm. As this characteristic signal does not feature in the parent complex without the thiophene bridge, it is attributed to delocalized ligand-based transitions involving the thiophene moiety.^39^ This is also consistent with the energy and character of the TDDFT-predicted T_9_ state, which is highly delocalized over the ligand system (CDD in Figure 4). The modes at 1173 and 1574 cm^–1^ are likely attributed to TPA^•+^ features,^39,81,83,96^ while the remaining modes are likely delocalized ligand and/or thiophene modes. Furthermore, the TR^3^ spectra of **Pt-thio-TPA** (Figure S10) are almost identical to those of **Re-thio-TPA**, emphasizing the ligand-based nature of the excited state and the lack of involvement of the metal center.

### Photophysics of M-CC-TPA

The possibility that the biexponential excited state decay could be due to the presence of different rotamers was further addressed by examining an additional ligand system containing an ethynyl linker between the phen and TPA moieties (Figure 1). This bridge also promotes communication between the donor and acceptor in a similar manner to the thiophene unit^38,57^ but maintains a rigid, planar system thereby avoiding ligand-based rotamers. The photophysical behavior of the Re(I) and Pt(II) ethynyl complexes, denoted **Re-CC-TPA** and **Pt-CC-TPA**, in the FC region is very similar to that of the thiophene species. Steady-state absorption and resonance Raman spectroscopy reveal that ILCT states, with significant bridge involvement, dominate the FC photophysics (Figures S11–S13 and Tables S21–S22). In the excited state, the photophysical properties are also very similar to the thiophene complexes. Like the thiophene complexes, the emission of the ethynyl complexes was too weak or short-lived for reliable kinetic emission measurements to be taken on our ns system. However, the TA spectra have significant excited state absorption at 476 as well as at ∼800 nm, with minimal differences observed between **Re-CC-TPA** and **Pt-CC-TPA** (Figure S14). The TA spectra are also successfully simulated, with the DFT-optimized triplet state possessing both ^3^ILCT and ^3^LC characters. Finally, long-lived biexponential decay from dark states was also observed, with lifetime components of 12.4 and 43.6 μs for **Re-CC-TPA**, and 11.2 and 31.7 μs for **Pt-CC-TPA**, with approximate τ_1_/τ_2_ weightings of 1:3 and 1:2, respectively. Akin to the thiophene complexes, larger SOC values are observed between upper singlet and triplet states of MLCT character, while the two lowest-lying triplet states retain ^3^ILCT/^3^LC character (Tables S23 and S24). These findings strongly suggest that for electronically conducting bridge species, the excited state decay is attributed to the presence of two noninteracting dark states delocalized over the ligand system and not to decay from different rotamers. Unfortunately, TR^3^ spectra could not be obtained for the **Re-CC-TPA** and **Pt-CC-TPA** complexes as the strong excited state absorption occurs at 476 nm (Figure S14), compared to the 515 nm absorption of the thiophene species and is therefore not in resonance with the 532.0 nm probe pulse.

### Photophysics of **Re-trz-TPA**

The importance of a conductive bridge between the TPA donor and phen acceptor to establish a long-lived excited state has been clearly demonstrated. As a point of contrast, the photophysical properties of a Re(I) complex with a triazole bridging group, denoted **Re-trz-TPA**, were also investigated, and four ground-state rotamers were identified (Table S25). As discussed previously, triazole groups—despite their aromatic nature—are well known to function as electronic insulators and can thereby disrupt donor–acceptor communication and, thus, the population of the desired long-lived charge-separated (e.g., ^3^ILCT) states.^56,57,61,97^ As anticipated, the presence of the triazole group slightly blue-shifts the electronic absorption spectrum relative to the thiophene and ethynyl complexes, with a λ_max_ of 392 nm and decreases the molar absorptivity (Figure S15). TDDFT calculations predict that, unlike the other complexes, the S_1_ state is MLCT in nature but with minimal oscillator strength (Tables S26–S29). Resonance Raman spectra of **Re-trz-TPA** are notably different from the other species, with the totally symmetric CO stretching mode at 2026 cm^–1^ showing appreciable enhancement at low excitation energies, indicative of the presence of such a low-lying MLCT state. This trend was also successfully replicated in the Boltzmann weighted simulated resonance Raman spectra (Figure S16).

Additional differences are also observed in the excited state properties. Unlike the thiophene and ethynyl complexes, the excited state absorption of **Re-trz-TPA** is weak and poorly resolved (Figure S17) and possesses a comparatively short lifetime of approximately 60 ns. In addition, unlike the other complexes that exhibited very weak and/or short emission, **Re-trz-TPA** emits at around 600 nm. This emissive lifetime is equivalent to that obtained in the TA measurements, implying that the same state is responsible for both signals. Furthermore, photocounting measurements of the ligand showed that, unlike thio-TPA, the ligand emission decay of trz-TPA was monoexponential (Figure S8). Four triplet ground-state rotamers of **Re-trz-TPA** were identified, and analysis of the spin density of each **Re-trz-TPA** rotamer reveals that the triplet state is ^3^MLCT in nature, in contrast to the ^3^ILCT/^3^LC states of thiophene and ethynyl complexes. Furthermore, the triplet geometries are significantly less planar, which is in line with the different nature of the triplet state between the complexes with insulating vs conducting bridges. SR calculations show coupling values of around 500 cm^–1^ between singlet and triplet MLCT states of similar energy within the FC geometry (Table S30). The differing photophysical properties, namely, the ^3^MLCT character and shorter excited state lifetime of this insulating-bridge complex highlight the importance of the presence of low-lying ^3^ILCT/^3^LC states (populated via MLCT gateway states) to achieve desired long lifetimes.

## Conclusions

A thorough spectroscopic and computational investigation has been performed to understand the photophysical behavior exhibited by a series of bichromophoric complexes. Steady-state techniques combined with DFT and TDDFT simulations allowed the underlying FC photophysics to be investigated, while the nature of the excited state relaxation pathways was elucidated with time-resolved techniques in conjunction with (SR)-TDDFT. The nature of the bridging group has been shown to dominate the excited state photophysics in the investigated Re(I) and Pt(II) complexes. Incorporating an electronically conducting thiophene or ethynyl unit facilitates the generation of strongly absorbing dark states with long biexponential lifetimes. The presence of rotamers in solution was identified, but based on the combination of the aforementioned techniques and a careful comparison of the predicted behavior of each rotamer, the biexponential excited state decay was attributed to the presence of two highly delocalized ^3^ILCT/^3^LC states and not to separate rotamers. Such long-lived states have not been observed in previous studies involving phen-TPA complexes,^39,63^ or even within similar dppz-B-TPA complexes incorporating the same bridging units.^38,42^

These findings illustrate how the use of a bichromophoric molecule, with both metal and organic components, can be utilized to successfully generate long-lived excited states. Strong SOC facilitates ^3^MLCT population, but if low energy ^3^ILCT/^3^LC states are also present (achieved via incorporation of a conducting bridge species), these are subsequently populated via internal conversion. We therefore highlight the importance of energetic and electronic matching of the structural components to achieve prolongated excited states and that although click-chemistry is a strong tool for the synthesis of triazoles, the use of such electronically insulating bridging groups should be avoided if long-lived excited states are the desired outcome. Such long-lived triplet states have the potential to be used in additional electron and/or energy transfer steps, such as within photocatalytic reaction processes, e.g., in the context of clean energy generation, to allow the activation of the catalytic unit, either in a heterogeneous or homogeneous fashion.

## Supporting Information Available

Additional structural and energetic information on the rotamers, as well as a range of experimental and simulated spectra, SOC tables, and additional synthetic, spectroscopic, and computational details; *xyz* structures can be found at 10.5281/zenodo.10102441 free of charge (PDF)
