# Supplementary material for: Controlling Excited State Localization in Bichromophoric Photosensitizers via the Bridging Group
Source: Inorg Chem. 2024 Mar 4;63(11):4947–56. doi: 10.1021/acs.inorgchem.3c04110 (PMC10951951; doi:10.1021/acs.inorgchem.3c04110)
Supplement: Supplementary file 1 — ic3c04110_si_001.pdf [file ic3c04110_si_001.pdf]

Controlling excited state localisation in bichromophoric photosensitisers via the bridging group

Georgina E. Shillito[a*], Dan Preston[b], James D. Crowley[c,d], Pawel Wagner[e], Samuel J. Harris[c,d], Keith C. Gordon[c,d], and Stephan Kupfer[a*]

[a] Institute of Physical Chemistry, Friedrich Schiller University Jena,
Helmholtzweg 4, 07743 Jena, Germany
*E-mail: georgina.shillito@uni-jena.de
*E-mail: stephan.kupfer@uni-jena.de

[b] Research School of Chemistry, Australian National University,
Canberra ACT 2600, Australia

[c] Department of Chemistry, University of Otago,
362 Leith Street, Dunedin 9016, New Zealand

[d] MacDiarmid Institute for Advanced Materials and Nanotechnology,
Wellington 6012, New Zealand

[e] University of Wollongong, Wollongong,
Northfields Ave Wollongong, NSW 2522 Australia

Supporting Information

Section 1 – Additional figures and tables

Section 2 – Experimental

- Ligand synthesis
- Complex synthesis
- Spectroscopy
- Computational details

Section 1: Additional figures and tables

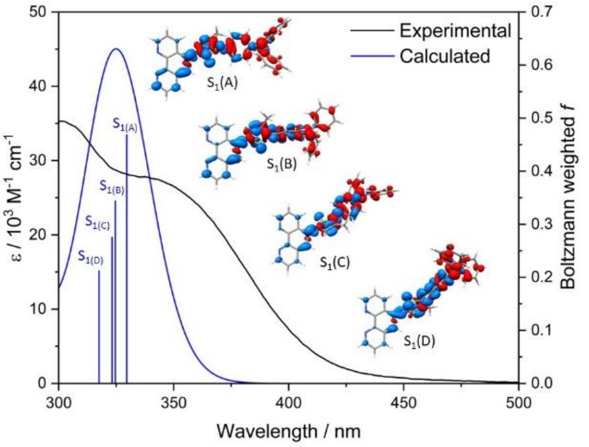

Figure S1. Experimental (black line) and the Boltzmann weighted TD-DFT (CAM-B3LYP, def2-SVP, CH_2Cl_2 solvent field) (blue line) calculated electronic absorption spectra of **thio-TPA**. The transition energies for the S_1 state (ILCT) for each rotamer (A-D) are labelled and their intensities scaled by their Boltzmann weighted population. The charge density difference (CDD) (red to blue) for this state is shown for each rotamer.

Table S1. Rotamers of **thio-TPA** and the corresponding Re(I) and Pt(II) complexes in both singlet and triplet multiplicities. The rotamers are labelled A-D with respect to increasing energy relative to the lowest identified S_0 state. The measured dihedral angle is indicated in red in the corresponding structure.

thio-TPA		
Label	Dihedral Angle / °	Relative energy / eV
	S_0	S_0
a	-47.5	0.0000
b	47.5	0.0069
c	122.5	0.0157
d	-122.5	0.0204

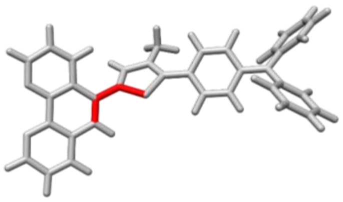

Re-thio-TPA				
Label	Dihedral Angle / °		Relative energy / eV	
	S_0	T_1	S_0	T_1
a	-45.0	-170.0	0.0000	1.9046
b	45.0	170.4	0.0072	1.9088
c	125.1	15.4	0.0204	1.9385
d	-125.0	-14.6	0.0278	1.9423

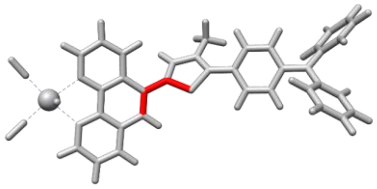

Pt-thio-TPA				
Label	Dihedral Angle / °		Relative energy / eV	
	S_0	T_1	S_0	T_1
a	-45.8	-170.4	0.0000	1.9030
b	44.2	169.6	0.0085	1.9050
c	129.2	-15.4	0.0207	1.9368
d	-125.8	14.6	0.0292	1.9393

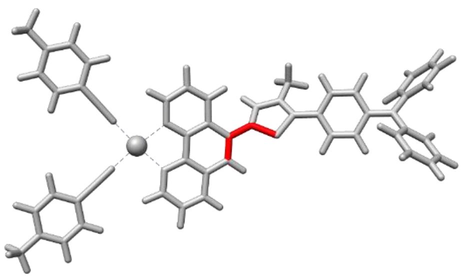

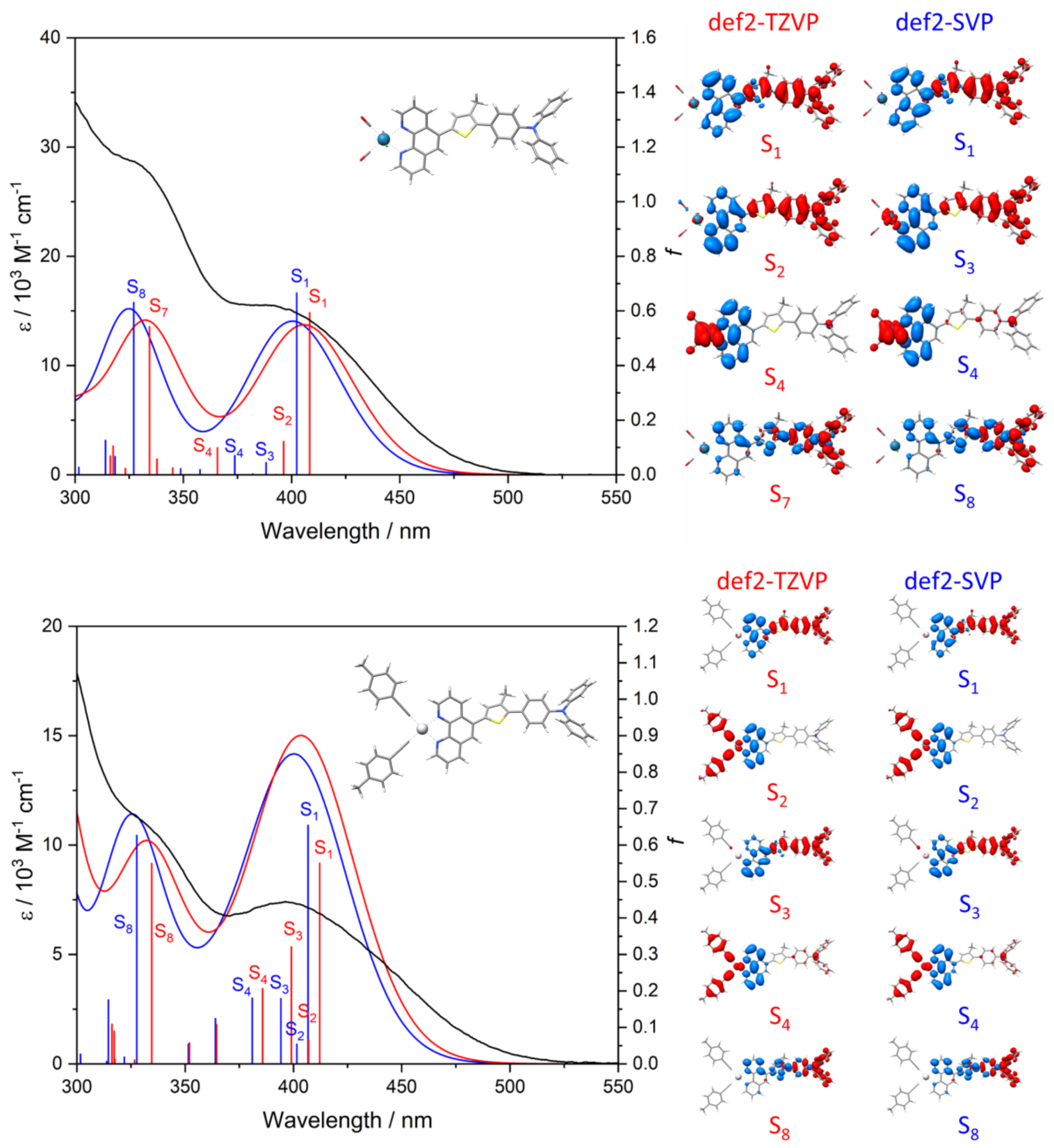

Figure S2. TDDFT calculations performed on the lowest energy rotamer of **Re-thio-TPA** (upper) and **Pt-thio-TPA** (lower) using the def2-TZVP (red) and def2-SVP (blue) basis sets. The charge density differences (CDDs) are given on the right-hand side for the key states (electron density transferred from red to blue).

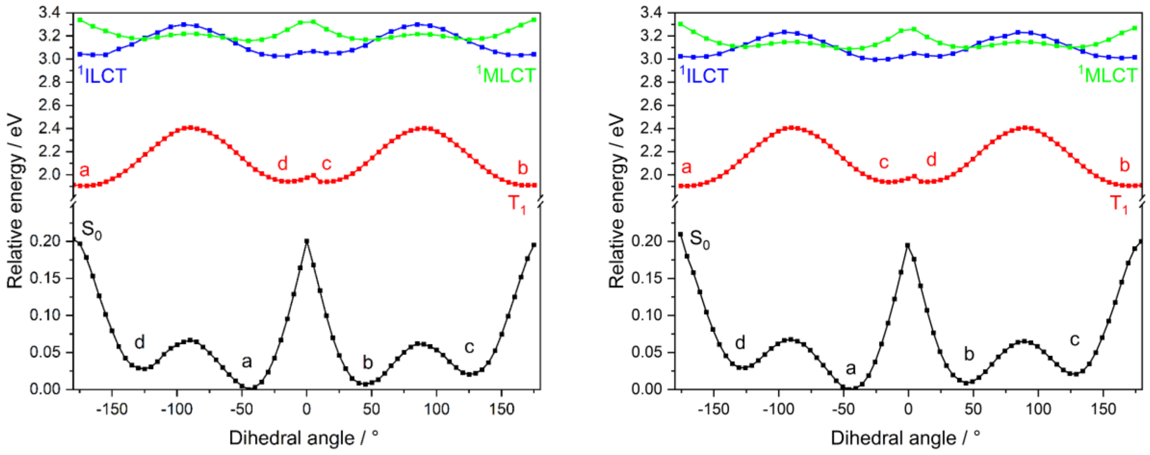

Figure S3. Energies of the ground state singlet (black) and triplet (red) states with respect to variation of the bridge dihedral angle obtained from singlet and triplet DFT relaxed scans. The minima on the potential energy surfaces correspond to the four S_0 and T_1 rotamers identified and are labelled A-D, with A being the lowest in energy. The energies of the ^{1}MLCT and ^{1}ILCT states were obtained by TDDFT calculations from the respective S_0 geometries. Left: **Re-thio-TPA**; Right: **Pt-thio-TPA.**

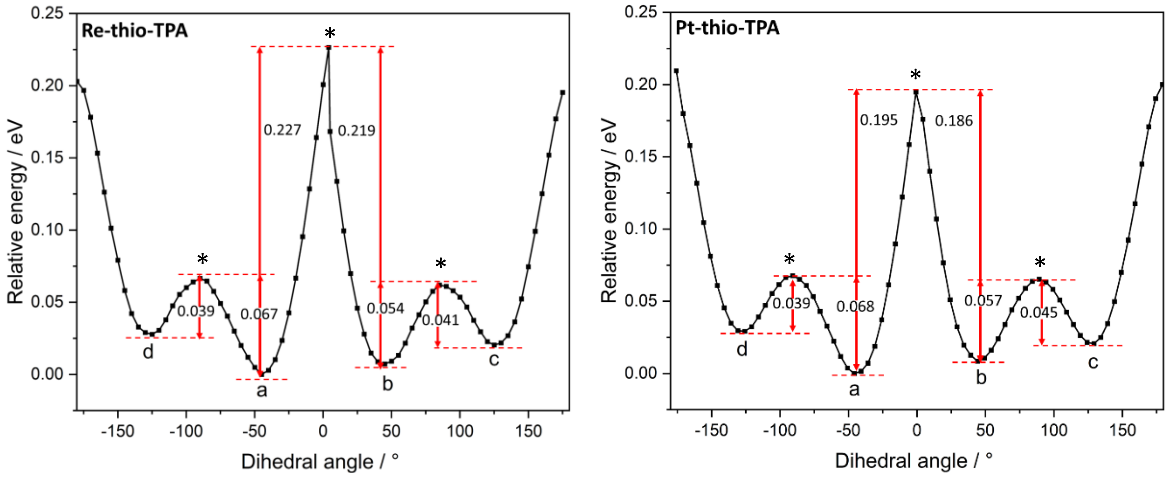

Figure S4. Ground state (black) potential energy surface with respect to variation of the bridge dihedral angle obtained from DFT relaxed scans. The minima correspond to the four S_0 rotamers identified and are labelled A-D, with A being the lowest in energy. Transition states are shown at the marked * points, whose structures were identified through the presence of a single negative frequency. The respective barrier heights are indicated in eV. Left: **Re-thio-TPA**; Right: **Pt-thio-TPA**.

Table S2. TDDFT calculations of **Re-thio-TPA** (rotamer A) detailing the lowest eight singlet states obtained from the FC geometry using the def2-TZVP basis set. CDDs are given for each transition (red→blue) and the molecular orbitals are shown below in blue and green.

**Re-thio-TPA Rotamer A (def2-TZVP)**	E / eV	λ / nm	*f*	Wgt %	From	To	CDD
**S_1**	3.04	408	0.593	38<br>46	173<br>173	174<br>175	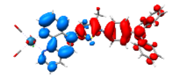
**S_2**	3.13	396	0.122	48<br>40	173<br>173	174<br>175	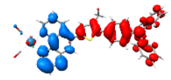
**S_3**	3.25	382	0.003	97	172	174	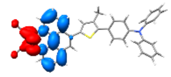
**S_4**	3.39	366	0.099	30<br>61	170<br>171	174<br>174	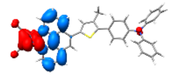
**S_5**	3.59	345	0.025	94	172	175	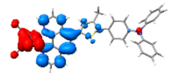
**S_6**	3.67	338	0.058	20<br>69	170<br>171	175<br>175	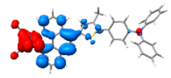
**S_7**	3.71	334	0.542	74	173	176	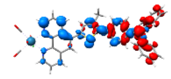
**S_8**	3.72	333	0.000	93	169	174	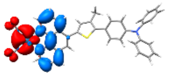

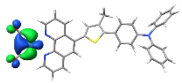
169

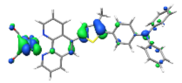
170

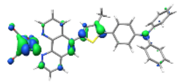
171

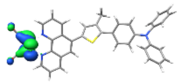
172

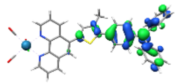
173

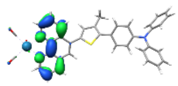
174

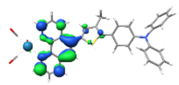
175

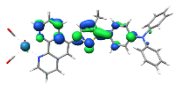
176

Table S3. TDDFT calculations of **Re-thio-TPA** (rotamer A) detailing the lowest eight singlet states obtained from the FC geometry using the def2-SVP basis set. CDDs are given for each transition (red→blue) and the molecular orbitals are shown below in blue and green.

**Re-thio-TPA Rotamer A**	E / eV	λ / nm	*f*	Wgt %	From	To	CDD
**S_1**	3.08	402	0.6653	34<br>51	173<br>173	174<br>175	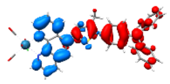
**S_2**	3.16	393	0.0007	92	172	174	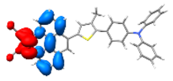
**S_3**	3.19	388	0.0445	11<br>44<br>36	171<br>173<br>173	174<br>174<br>175	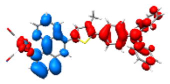
**S_4**	3.32	374	0.0700	75<br>11	171<br>173	174<br>174	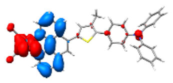
**S_5**	3.47	358	0.0185	92	172	175	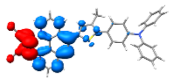
**S_6**	3.56	349	0.0220	89	171	175	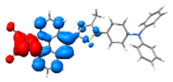
**S_7**	3.74	331	0.0002	93	169	174	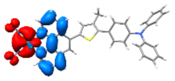
**S_8**	3.79	327	0.6305	80	173	176	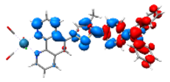

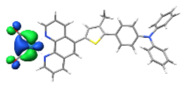
169

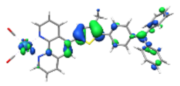
170

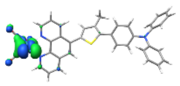
171

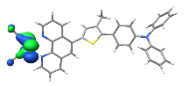
172

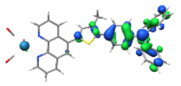
173

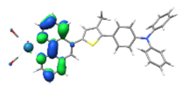
174

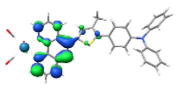
175

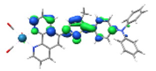
176

Table S4. TDDFT calculations of **Re-thio-TPA** (rotamer B) detailing the lowest eight singlet states obtained from the FC geometry using the def2-SVP basis set. CDDs are given for each transition (red→blue) and the molecular orbitals are shown below in blue and green.

**Re-thio-TPA Rotamer B**	E / eV	λ / nm	*f*	Wgt %	From	To	CDD
**S_1**	3.11	398	0.5279	24<br>59	173<br>173	174<br>175	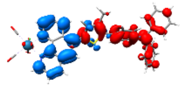
**S_2**	3.16	392	0.0126	95	172	174	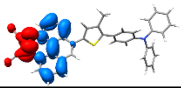
**S_3**	3.22	385	0.0237	15<br>52<br>26	171<br>173<br>173	174<br>174<br>175	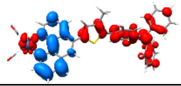
**S_4**	3.31	374	0.0849	73<br>15	171<br>173	174<br>174	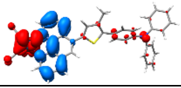
**S_5**	3.47	357	0.0216	94	172	175	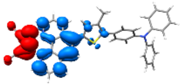
**S_6**	3.55	349	0.0252	91	171	175	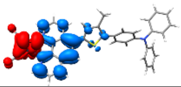
**S_7**	3.74	332	0.0013	92	169	174	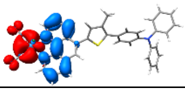
**S_8**	3.80	326	0.7181	79<br>9	173<br>173	176<br>177	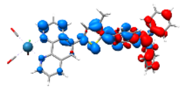

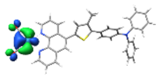	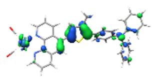	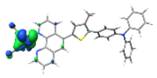	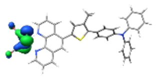	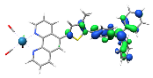
169	170	171	172	173
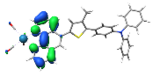	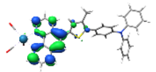	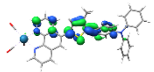	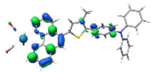	
174	175	176	177	

Table S5. TDDFT calculations of **Re-thio-TPA** (rotamer C) detailing the lowest eight singlet states obtained from the FC geometry using the def2-SVP basis set. CDDs are given for each transition (red→blue) and the molecular orbitals are shown below in blue and green.

**Re-thio-TPA Rotamer C**	E / eV	λ / nm	*f*	Wgt %	From	To	CDD
**S_1**	3.13	396	0.4457	37<br>46	173<br>173	174<br>175	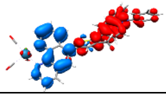
**S_2**	3.15	394	0.0068	96	172	174	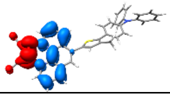
**S_3**	3.22	385	0.1783	8<br>43<br>40	171<br>173<br>173	174<br>174<br>175	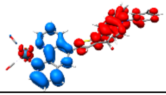
**S_4**	3.30	376	0.0918	81<br>11	171<br>173	174<br>174	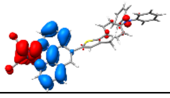
**S_5**	3.48	357	0.0186	95	172	175	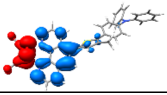
**S_6**	3.56	348	0.0179	94	171	175	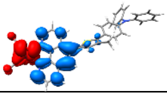
**S_7**	3.73	332	0.0025	95	169	174	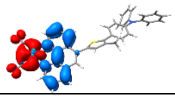
**S_8**	3.77	329	0.6463	81	173	176	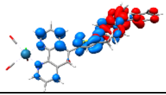

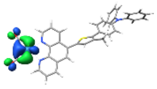
169

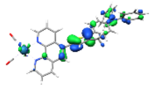
170

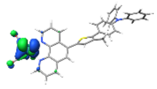
171

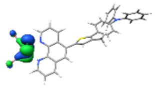
172

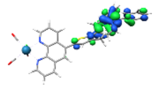
173

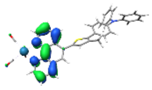
174

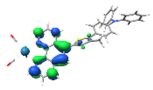
175

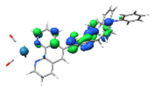
176

Table S6. TDDFT calculations of **Re-thio-TPA** (rotamer D) detailing the lowest eight singlet states obtained from the FC geometry using the def2-SVP basis set. CDDs are given for each transition (red→blue) and the molecular orbitals are shown below in blue and green.

**Re-thio-TPA Rotamer D**	E / eV	λ / nm	*f*	Wgt %	From	To	CDD
**S_1**	3.15	394	0.0550	78<br>9<br>8	172<br>173<br>173	174<br>174<br>175	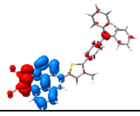
**S_2**	3.16	393	0.2512	19<br>38<br>29	172<br>173<br>173	174<br>174<br>175	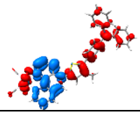
**S_3**	3.25	382	0.1031	12<br>31<br>49	171<br>173<br>173	174<br>174<br>175	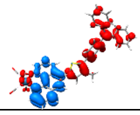
**S_4**	3.30	376	0.1061	77<br>15	171<br>173	174<br>174	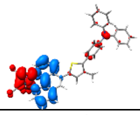
**S_5**	3.48	356	0.0207	96	172	175	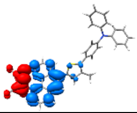
**S_6**	3.57	348	0.0233	94	171	175	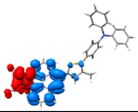
**S_7**	3.73	332	0.0017	93	169	174	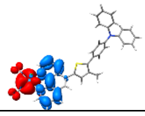
**S_8**	3.77	329	0.8193	79<br>8	173<br>173	176<br>177	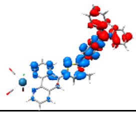

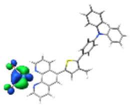
169

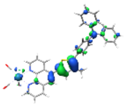
170

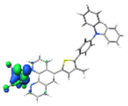
171

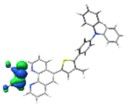
172

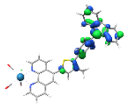
173

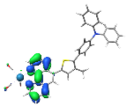
174

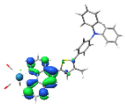
175

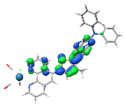
176

Table S7. TDDFT calculations of **Pt-thio-TPA** (rotamer A) detailing the lowest eight singlet states obtained from the FC geometry using the def2-TZVP basis set. CDDs are given for each transition (red→blue) and the molecular orbitals are shown below in blue and green.

**Pt-thio-TPA Rotamer A (def2-TZVP)**	E / eV	λ / nm	*f*	Wgt %	From	To	CDD
**S_1**	3.01	412	0.550	58<br>22	206<br>206	207<br>208	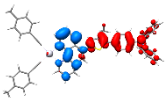
**S_2**	3.05	407	0.065	92	205	207	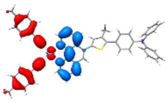
**S_3**	3.11	399	0.321	22<br>61	206<br>206	207<br>208	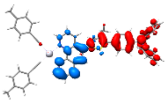
**S_4**	3.21	386	0.206	78<br>11	204<br>206	207<br>207	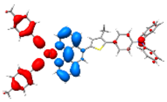
**S_5**	3.40	365	0.108	90	205	208	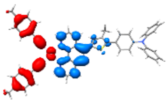
**S_6**	3.52	352	0.057	94	204	208	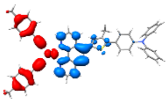
**S_7**	3.67	338	0.000	96	202	207	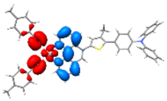
**S_8**	3.71	335	0.550	77	206	209	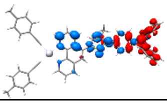

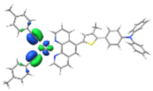

202

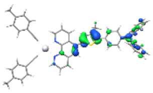

203

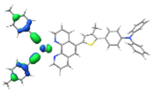

204

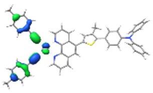

205

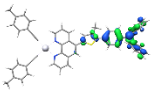

206

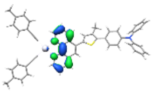

207

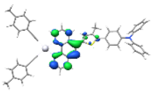

208

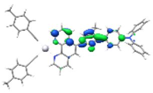

209

Table S8. TDDFT calculations of **Pt-thio-TPA** (rotamer A) detailing the lowest eight singlet states obtained from the FC geometry using the def2-SVP basis set. CDDs are given for each transition (red→blue) and the molecular orbitals are shown below in blue and green.

**Pt-thio-TPA Rotamer A**	E / eV	λ / nm	f	Wgt %	From	To	CDD
**S_1**	3.05	407	0.6540	49<br>33	206<br>206	207<br>208	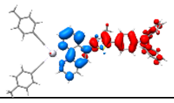
**S_2**	3.09	402	0.0540	93	205	207	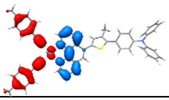
**S_3**	3.15	394	0.1789	31<br>52	206<br>206	207<br>208	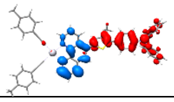
**S_4**	3.25	381	0.1802	74<br>10<br>11	204<br>205<br>206	207<br>208<br>207	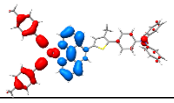
**S_5**	3.41	364	0.1237	11<br>86	204<br>205	207<br>208	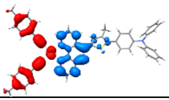
**S_6**	3.53	351	0.0540	94	204	208	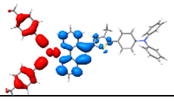
**S_7**	3.72	333	0.0000	96	202	207	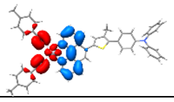
**S_8**	3.78	328	0.6265	78<br>8	206<br>206	209<br>210	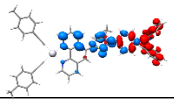

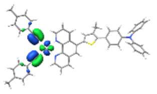
202
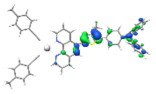
203
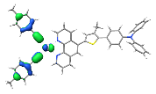
204
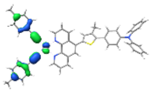
205
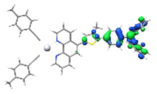
206
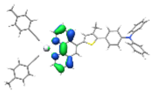
207
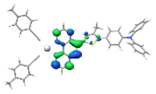
208
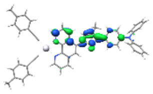
209
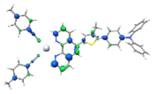
210

Table S9. TDDFT calculations of **Pt-thio-TPA** (rotamer B) detailing the lowest eight singlet states obtained from the FC geometry using the def2-SVP basis set. CDDs are given for each transition (red→blue) and the molecular orbitals are shown below in blue and green.

**Pt-thio-TPA Rotamer B**	E / eV	λ / nm	*f*	Wgt %	From	To	CDD
**S_1**	3.08	403	0.5463	41<br>40	206<br>206	207<br>208	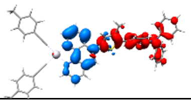
**S_2**	3.09	401	0.0593	94	205	207	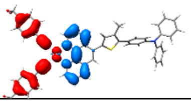
**S_3**	3.16	392	0.1139	36<br>46	206<br>206	207<br>208	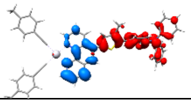
**S_4**	3.25	382	0.2010	73<br>9<br>14	204<br>205<br>206	207<br>208<br>207	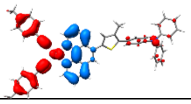
**S_5**	3.41	364	0.1308	10<br>87	204<br>205	207<br>208	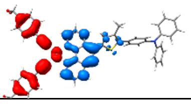
**S_6**	3.52	352	0.0541	95	204	208	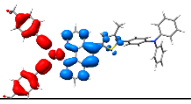
**S_7**	3.72	333	0.0092	94	202	207	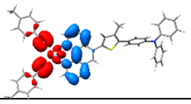
**S_8**	3.80	326	0.7276	73<br>10	206<br>206	209<br>210	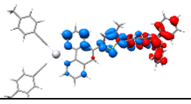

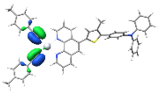

202

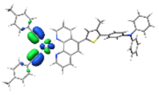

203

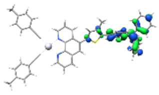

204

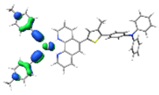

205

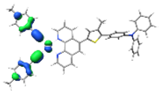

206

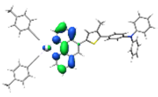

207

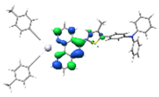

208

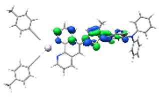

209

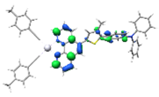

210

Table S10. TDDFT calculations of **Pt-thio-TPA** (rotamer C) detailing the lowest eight singlet states obtained from the FC geometry using the def2-SVP basis set. CDDs are given for each transition (red→blue) and the molecular orbitals are shown below in blue and green.

**Pt-thio-TPA Rotamer C**	E / eV	λ / nm	*f*	Wgt %	From	To	CDD
**S_1**	3.07	404	0.4132	58<br>25	206<br>206	207<br>208	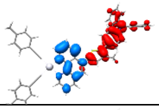
**S_2**	3.08	402	0.0526	95	205	207	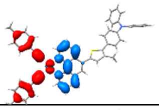
**S_3**	3.16	392	0.3825	25<br>61	206<br>206	207<br>208	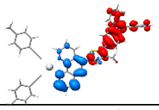
**S_4**	3.24	383	0.2143	78<br>10	204<br>206	207<br>207	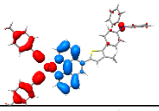
**S_5**	3.41	364	0.1200	9<br>88	204<br>205	207<br>208	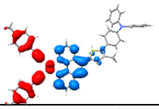
**S_6**	3.53	351	0.0463	95	204	208	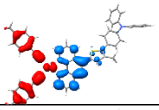
**S_7**	3.71	334	0.0065	95	202	207	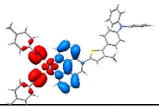
**S_8**	3.78	328	0.6001	77	206	209	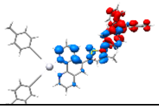

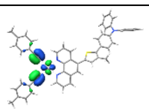<br>202	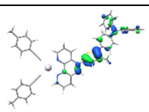<br>203	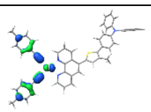<br>204	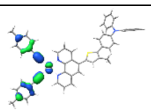<br>205	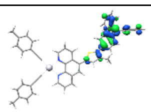<br>206
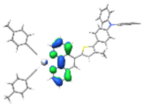<br>207	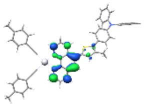<br>208	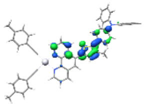<br>209		

Table S11. TDDFT calculations of **Pt-thio-TPA** (rotamer D) detailing the lowest eight singlet states obtained from the FC geometry using the def2-SVP basis set. CDDs are given for each transition (red→blue) and the molecular orbitals are shown below in blue and green.

**Pt-thio-TPA Rotamer D**	E / eV	λ / nm	*f*	Wgt %	From	To	CDD
**S_1**	3.08	403	0.0634	95	205	207	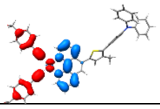
**S_2**	3.10	400	0.2781	65<br>18	206<br>206	207<br>208	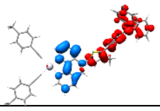
**S_3**	3.20	387	0.2061	15<br>68	206<br>206	207<br>208	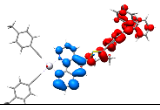
**S_4**	3.24	383	0.2595	74<br>14	204<br>206	207<br>207	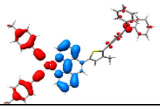
**S_5**	3.42	363	0.1291	8<br>89	204<br>205	207<br>208	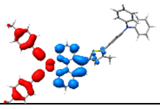
**S_6**	3.54	350	0.0511	95	204	208	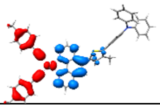
**S_7**	3.71	334	0.0000	96	202	207	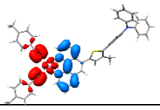
**S_8**	3.76	329	0.7887	77<br>9	206<br>206	209<br>210	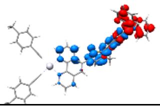

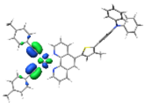	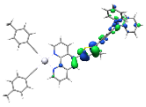	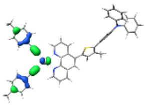	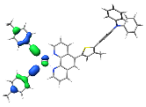	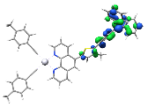
202	203	204	205	206
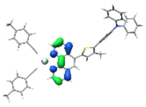	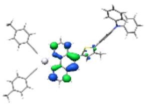	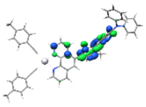	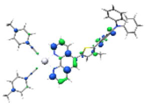	
207	208	209	210	

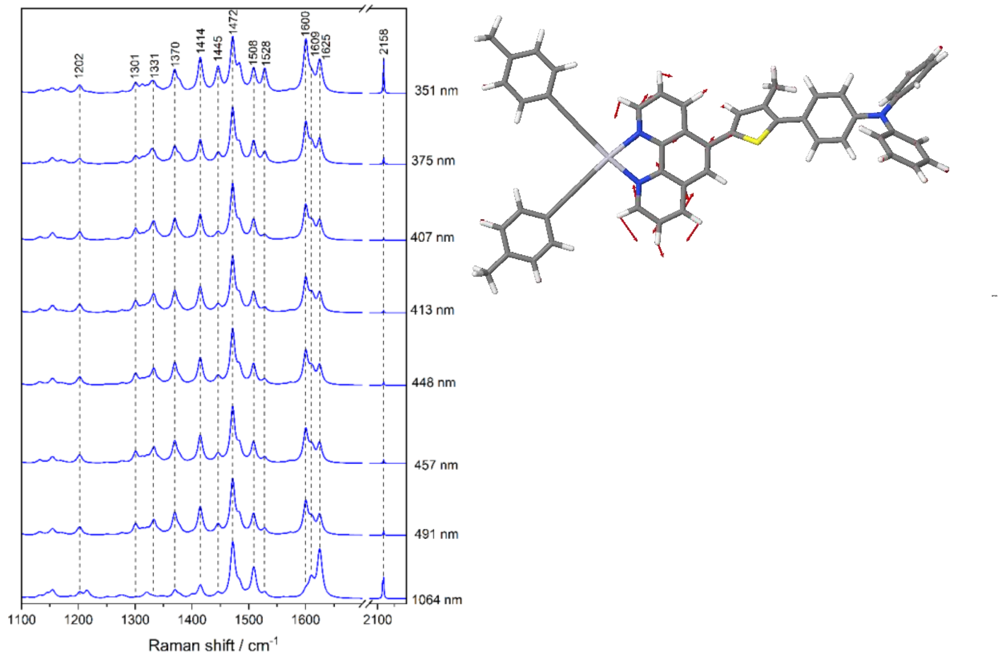

Figure S5. Left: Calculated resonance Raman spectra of **Pt-thio-TPA** utilising Boltzmann weighted contributions from the four S_0 rotamers, frequencies are scaled by 0.95. Right: Vibrational mode #235 (scaled frequency = 1600 cm^{-1}).

Table S12. Molecular orbitals involved in the two lowest energy ILCT states (S_1 and S_3) in **Re-thio-TPA** and **Pt-thio-TPA** using the def2-SVP basis set. The orbital composition is given from the lowest energy singlet rotamer (A).

Re-thio-TPA	#MO	Pt-thio-TPA	#MO
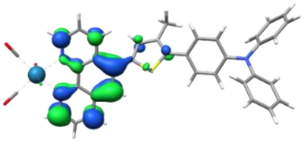	**175 (a_2)**	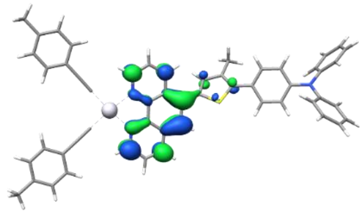	208 (a_2)
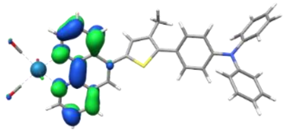	174 (b_1)	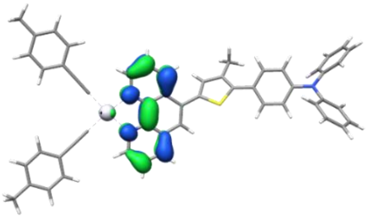	**207 (b_1)**
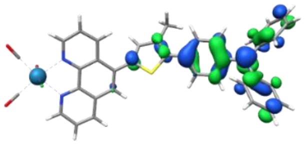	**173**	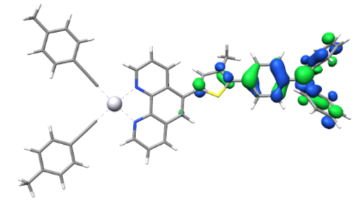	**206**
**S_1 (ILCT, 3.08 eV, f = 0.67)**	**%Weight**	**S_1 (ILCT, 3.05 eV, f = 0.65)**	**%Weight**
173 → 174	34	206 → 207	49
173 → 175	51	206 → 208	33
**S_3 (ILCT, 3.19 eV, f = 0.04)**	**%Weight**	**S_3 (ILCT, 3.15 eV, f = 0.18)**	**%Weight**
173 → 174	44	206 → 207	31
173 → 175	36	206 → 208	52
171→ 174	11	-	

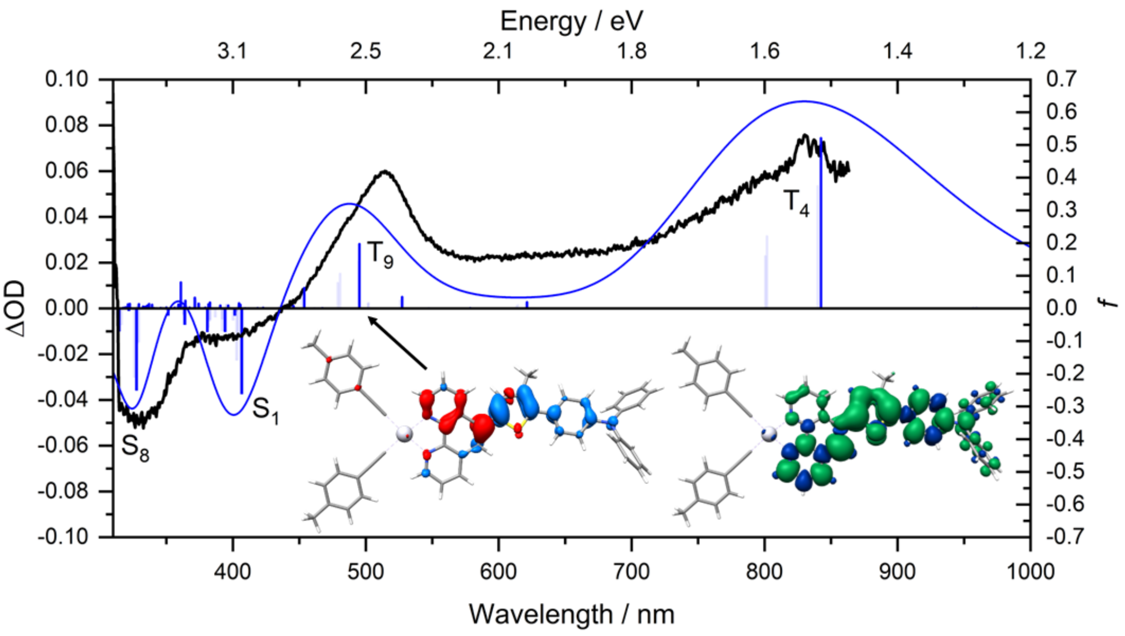

Figure S6. Boltzmann weighted simulated (blue - B3LYP35, def2-SVP, CH_2Cl_2 solvent field) and experimental (black) transient absorption spectra of **Pt-thio-TPA** obtained in degassed CH_2Cl_2. The downward vertical bars correspond to the ground state bleach (singlet-singlet transitions in the S_0 equilibrium) while upward vertical bars are associated with excited state absorption (triplet-triplet transitions in the T_1 structure). Bold bars correspond to rotamer A. The oscillator strengths have been scaled based on the Boltzmann weighting. The left inset shows the CDD for the T_9 state and the right inset shows the spin density distribution of the complex in the optimised T_1 geometry which would likely be obtained from excitation of the S_0 rotamer A.

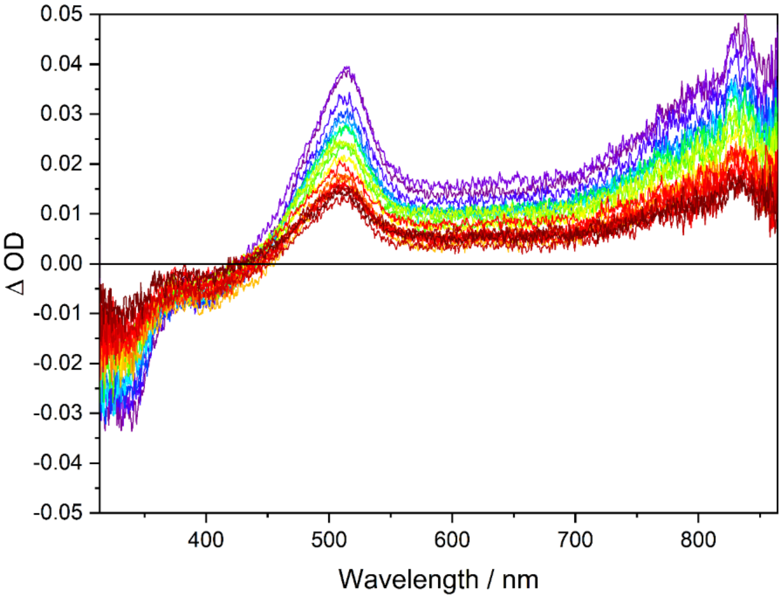

Figure S7. Transient absorption spectra of **Pt-thio-TPA** taken at 2 µs time intervals between delays of 0 (purple) and 40 µs (red) following initial excitation.

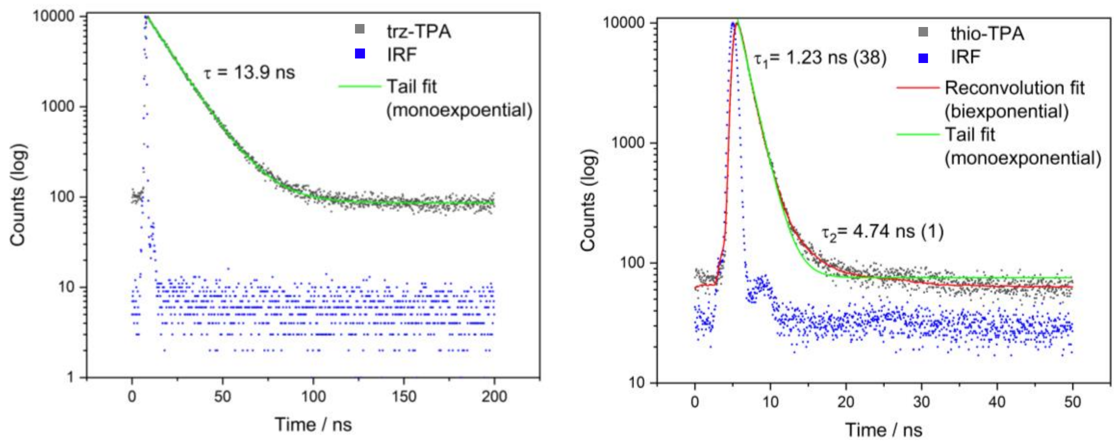

Figure S8: Photon counting lifetime measurements of the ligands thio-TPA and trz-TPA obtained at their respective emission wavelengths of 524 and 545 nm. The instrument response function (IRF) is measured with a Ludox solution. A monoexponential tail fit (green) was performed on trz-TPA while a biexponential fit was required to fit thio-TPA (red) a monoexponential fit (green) is also shown for comparison.

Table S13: Spin orbit couplings between the first four singlet and five triplet states of **Re-thio-TPA** (rotamer A - FC S_0 geometry) using the a) ZORA-def2-SVP and b) DKH-def2-SVP basis sets. The respective state energies and CDDs (red →blue) are from the TDDFT ORCA calculations.

**Re-thio-TPA: S_0 Rotamer A**			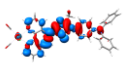	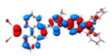	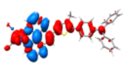	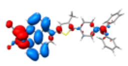	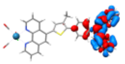
a) ZORA-def2-SVP		E / eV	2.24	2.66	2.89	3.01	3.13
	E / eV		**T_1**	**T_2**	**T_3**	**T_4**	**T_5**
	0.00	**S_0**	13	42	123	188	3
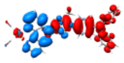	3.08	**S_1**	11	31	56	87	1
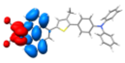	3.11	**S_2**	11	15	381	124	4
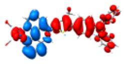	3.19	**S_3**	33	74	62	210	2
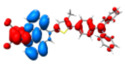	3.28	**S_4**	67	158	66	486	4

**Re-thio-TPA: S_0 Rotamer A**			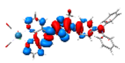	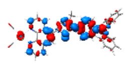	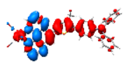	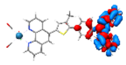	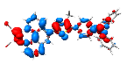
b) DKH-def2-SVP		E / eV	2.25	2.68	2.99	3.13	3.17
	E / eV		**T_1**	**T_2**	**T_3**	**T_4**	**T_5**
	0.00	**S_0**	8	22	76	3	95
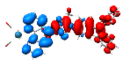	3.12	**S_1**	3	9	8	0	17
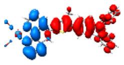	3.25	**S_2**	10	16	3	1	43
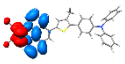	3.42	**S_3**	5	4	237	2	47
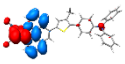	3.53	**S_4**	54	102	45	6	273

Table S14: Spin orbit couplings between the first four singlet and five triplet states of **Re-thio-TPA** (rotamer B - FC S_0 geometry) using the a) ZORA-def2-SVP and b) DKH-def2-SVP basis sets. The respective state energies and CDDs (red →blue) are from the TDDFT ORCA calculations.

Re-thio-TPA:
S_0 Rotamer B
a) ZORA-def2-SVP

			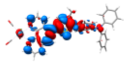	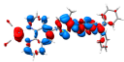	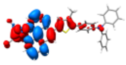	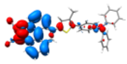	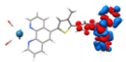
		E / eV	2.27	2.64	2.91	3.00	3.13
	E / eV		**T_1**	**T_2**	**T_3**	**T_4**	**T_5**
	0.00	**S_0**	15	41	140	198	1
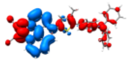	3.11	**S_1**	1	15	365	14	2
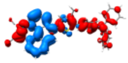	3.12	**S_2**	17	30	260	124	1
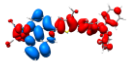	3.21	**S_3**	43	86	29	280	0
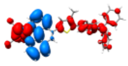	3.28	**S_4**	65	133	43	451	1

Re-thio-TPA:
S_0 Rotamer B
b) DKH-def2-SVP

			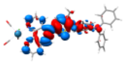	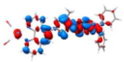	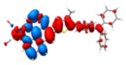	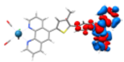	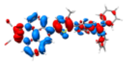
		E / eV	2.28	2.66	3.03	3.13	3.16
	E / eV		**T_1**	**T_2**	**T_3**	**T_4**	**T_5**
	0.00	**S_0**	10	22	91	3	95
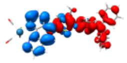	3.15	**S_1**	3	9	10	0	18
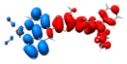	3.28	**S_2**	10	16	6	0	40
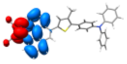	3.42	**S_3**	6	3	284	2	34
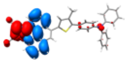	3.52	**S_4**	55	94	37	0	256

Table S15: Spin orbit couplings between the first four singlet and five triplet states of **Re-thio-TPA** (rotamer C – FC S_0 geometry) using the a) ZORA-def2-SVP and b) DKH-def2-SVP basis sets. The respective state energies and CDDs (red →blue) are from the TDDFT ORCA calculations.

Re-thio-TPA:
S_0 Rotamer C
a) ZORA-def2-SVP

			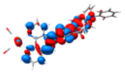	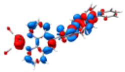	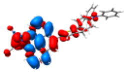	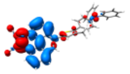	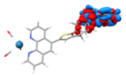
		E / eV	2.33	2.63	2.92	3.01	3.13
	E / eV		**T_1**	**T_2**	**T_3**	**T_4**	**T_5**
	0.00	**S_0**	19	40	146	197	6
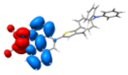	3.10	**S_1**	7	6	453	41	13
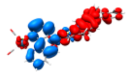	3.13	**S_2**	25	40	8	148	3
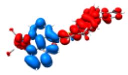	3.21	**S_3**	40	78	31	241	3
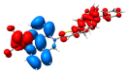	3.27	**S_4**	75	146	29	489	8

Re-thio-TPA:
S_0 Rotamer C
b) DKH-def2-SVP

			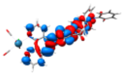	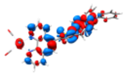	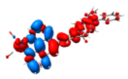	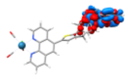	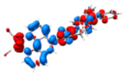
		E / eV	2.34	2.66	3.04	3.13	3.18
	E / eV		**T_1**	**T_2**	**T_3**	**T_4**	**T_5**
	0.00	**S_0**	12	21	93	4	93
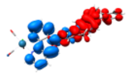	3.18	**S_1**	7	6	7	1	22
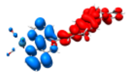	3.27	**S_2**	9	13	6	1	32
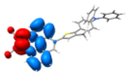	3.41	**S_3**	5	5	278	2	15
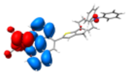	3.51	**S_4**	60	102	23	11	268

Table S16: Spin orbit couplings between the first four singlet and five triplet states of **Re-thio-TPA** (rotamer D – FC S_0 geometry) using the a) ZORA-def2-SVP and b) DKH-def2-SVP basis sets. The respective state energies and CDDs (red →blue) are from the TDDFT ORCA calculations.

Re-thio-TPA:
S_0 Rotamer D

a) ZORA-def2-SVP

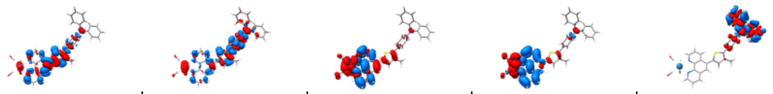

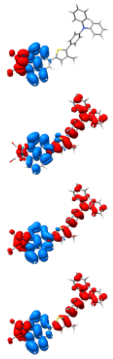

		E / eV	2.37	2.64	2.93	3.00	3.13
	E/ eV		**T_1**	**T_2**	**T_3**	**T_4**	**T_5**
	0.00	**S_0**	21	39	154	204	3
	3.10	**S_1**	5	5	483	60	7
	3.16	**S_2**	30	47	15	172	1
	3.24	**S_3**	69	103	39	369	3
	3.28	**S_4**	66	103	36	381	4

Re-thio-TPA:
S_0 Rotamer D

b) DKH-def2-SVP

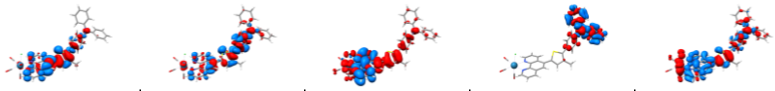

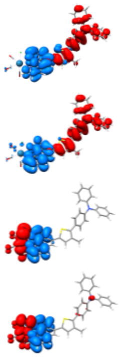

		E / eV	2.38	2.65	3.06	3.13	3.16
	E/ eV		**T_1**	**T_2**	**T_3**	**T_4**	**T_5**
	0.00	**S_0**	12	21	103	4	94
	3.20	**S_1**	6	10	11	0	18
	3.31	**S_2**	10	12	3	1	31
	3.41	**S_3**	5	2	310	7	62
	3.51	**S_4**	66	92	47	7	244

Table S17: Spin orbit couplings between the first five singlet and five triplet states of **Pt-thio-TPA** (rotamer A - FC S_0 geometry) using the a) ZORA-def2-SVP and b) DKH-def2-SVP basis sets. The respective state energies and CDDs (red →blue) are from the TDDFT ORCA calculations.

**Pt-thio-TPA: S_0 Rotamer A** a) ZORA-def2-SVP			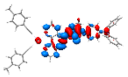	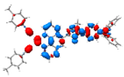	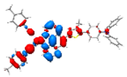	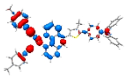	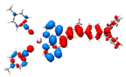
		E / eV	2.25	2.63	2.73	2.78	2.94
	E / eV		**T_1**	**T_2**	**T_3**	**T_4**	**T_5**
	0.00	**S_0**	15	53	16	33	14
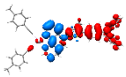	3.02	**S_1**	36	89	11	62	4
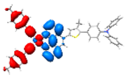	3.05	**S_2**	12	14	408	83	34
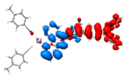	3.13	**S_3**	28	53	53	18	4
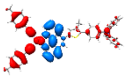	3.22	**S_4**	111	271	51	164	15
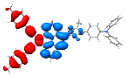	3.41	**S_5**	58	37	35	108	10

**Pt-thio-TPA: S_0 Rotamer A** b) DKH-def2-SVP			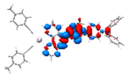	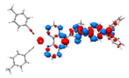	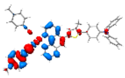	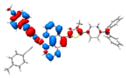	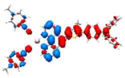
		E / eV	2.26	2.68	2.87	2.88	2.97
	E / eV		**T_1**	**T_2**	**T_3**	**T_4**	**T_5**
	0.00	**S_0**	6	19	9	7	9
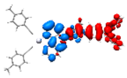	3.08	**S_1**	8	18	15	10	3
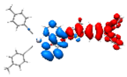	3.17	**S_2**	13	16	5	16	4
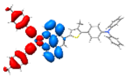	3.31	**S_3**	5	7	119	172	2
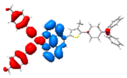	3.45	**S_4**	67	122	97	56	13
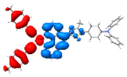	3.60	**S_5**	33	38	44	18	7

Table S18: Spin orbit couplings between the first five singlet and five triplet states of **Pt-thio-TPA** (rotamer B – FC S_0 geometry) using the a) ZORA-def2-SVP and b) DKH-def2-SVP basis sets. The respective state energies and CDDs (red →blue) are from the TDDFT ORCA calculations.

Pt-thio-TPA:
S_0 Rotamer B
a) ZORA-def2-SVP

			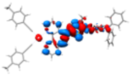	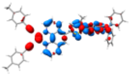	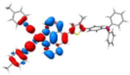	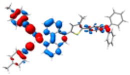	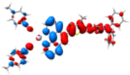
		E / eV	2.27	2.63	2.74	2.77	2.96
	E / eV		**T_1**	**T_2**	**T_3**	**T_4**	**T_5**
	0.00	**S_0**	18	49	13	36	15
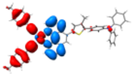	3.04	**S_1**	25	32	403	81	22
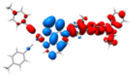	3.05	**S_2**	32	79	124	43	7
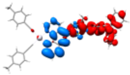	3.14	**S_3**	30	57	40	28	9
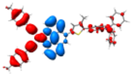	3.21	**S_4**	116	257	43	179	17
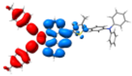	3.41	**S_5**	61	42	32	105	20

Pt-thio-TPA:
S_0 Rotamer B
b) DKH-def2-SVP

			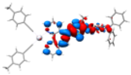	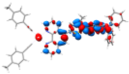	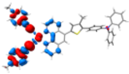	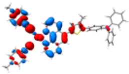	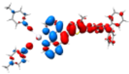
		E / eV	2.29	2.67	2.87	2.89	3.00
	E / eV		**T_1**	**T_2**	**T_3**	**T_4**	**T_5**
	0.00	**S_0**	9	17	11	7	11
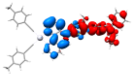	3.10	**S_1**	8	15	16	2	3
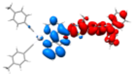	3.19	**S_2**	12	15	10	13	1
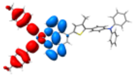	3.31	**S_3**	7	6	32	209	50
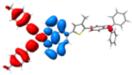	3.44	**S_4**	70	117	111	14	15
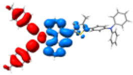	3.60	**S_5**	35	39	44	6	14

Table S19: Spin orbit couplings between the first five singlet and five triplet states of **Pt-thio-TPA** (rotamer C – FC S_0 geometry) using the a) ZORA-def2-SVP and b) DKH-def2-SVP basis sets. The respective state energies and CDDs (red→blue) are from the TDDFT ORCA calculations.

Pt-thio-TPA:
S_0 Rotamer C
a) ZORA-def2-SVP

			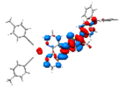	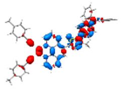	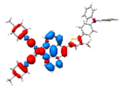	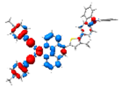	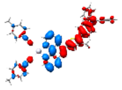
		E / eV	2.29	2.62	2.74	2.78	2.95
	E / eV		**T_1**	**T_2**	**T_3**	**T_4**	**T_5**
	0.00	**S_0**	20	51	10	36	10
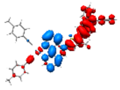	3.04	**S_1**	41	77	95	58	3
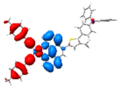	3.04	**S_2**	6	33	416	12	8
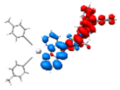	3.14	**S_3**	15	31	26	9	5
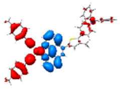	3.20	**S_4**	119	269	20	181	8
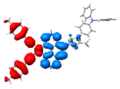	3.41	**S_5**	62	45	21	109	28

Pt-thio-TPA:
S_0 Rotamer C
b) DKH-def2-SVP

			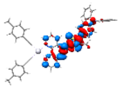	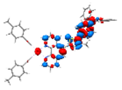	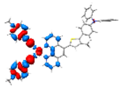	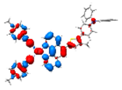	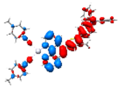
		E / eV	2.30	2.67	2.87	2.88	2.99
	E / eV		**T_1**	**T_2**	**T_3**	**T_4**	**T_5**
	0.00	**S_0**	10	17	9	10	8
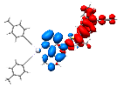	3.10	**S_1**	12	16	18	1	3
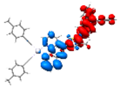	3.18	**S_2**	7	12	5	11	3
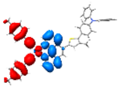	3.30	**S_3**	6	10	9	212	32
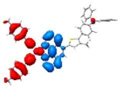	3.44	**S_4**	69	123	110	12	8
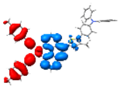	3.61	**S_5**	36	42	42	13	16

Table S20: Spin orbit couplings between the first five singlet and five triplet states of **Pt-thio-TPA** (rotamer D - FC S_0 geometry) using the a) ZORA-def2-SVP and b) DKH-def2-SVP basis sets. The respective state energies and CDDs (red →blue) are from the TDDFT ORCA calculations.

**Pt-thio-TPA:** **S_0 Rotamer D** a) ZORA-def2-SVP			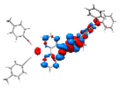	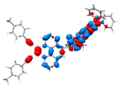	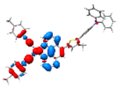	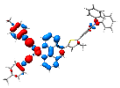	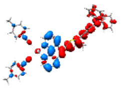
		E / eV	2.36	2.61	2.75	2.77	2.98
	E / eV		**T_1**	**T_2**	**T_3**	**T_4**	**T_5**
	0.00	**S_0**	22	47	10	38	13
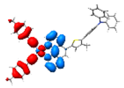	3.04	**S_1**	15	8	424	79	69
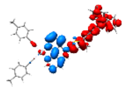	3.07	**S_2**	46	79	39	60	6
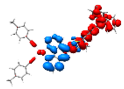	3.19	**S_3**	55	90	24	60	11
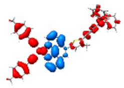	3.20	**S_4**	131	226	30	184	10
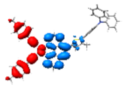	3.42	**S_5**	69	46	34	102	21

**Pt-thio-TPA:** **S_0 Rotamer D** b) DKH-def2-SVP			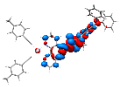	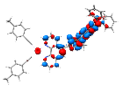	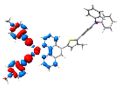	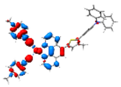	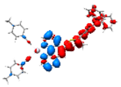
		E / eV	2.38	2.66	2.87	2.89	3.03
	E / eV		**T_1**	**T_2**	**T_3**	**T_4**	**T_5**
	0.00	**S_0**	9	17	7	7	10
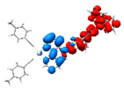	3.13	**S_1**	12	15	17	3	3
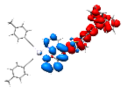	3.24	**S_2**	8	9	4	15	2
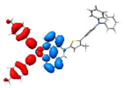	3.30	**S_3**	6	7	22	205	88
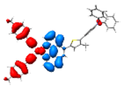	3.44	**S_4**	77	115	112	10	12
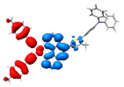	3.62	**S_5**	40	43	41	7	14

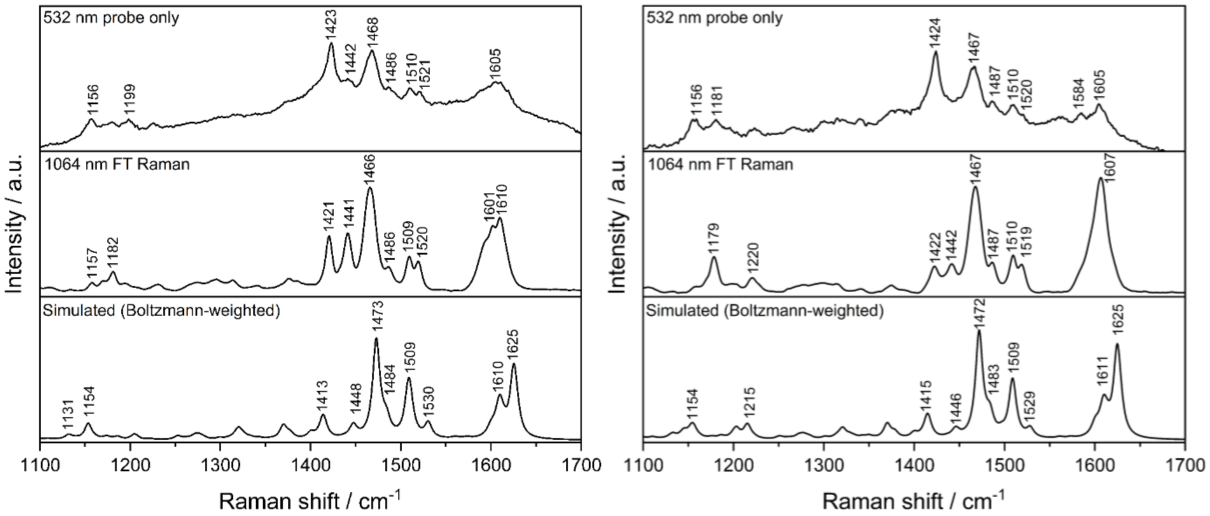

Figure S9. TR[3] probe only spectrum measured in degassed CH_2Cl_2, compared to the non-resonant 1064 nm spectrum and the DFT simulated non-resonant spectrum at 532 nm. Left: **Re-thio-TPA**; right: **Pt-thio-TPA**.

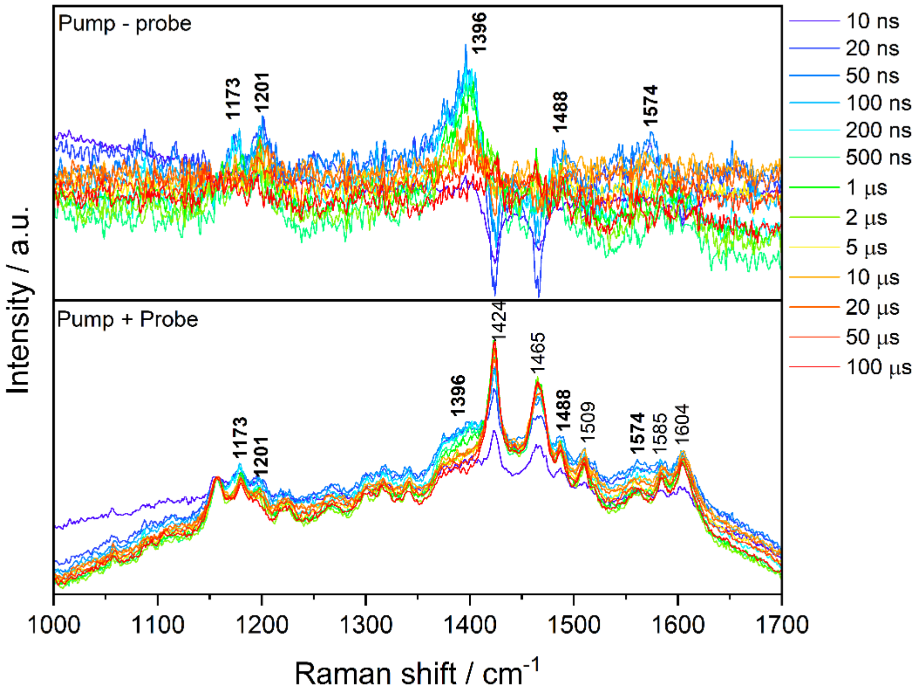

Figure S10. TR[3] spectra of **Pt-thio-TPA** obtained with pump (354.7 nm) probe (532.0 nm) delays between 10 ns and 100 μs in degassed CH_2Cl_2. The lower panel shows the pump + probe spectra normalised to the 1157 cm^{-1} solvent band, while the upper panel shows the result of subtraction of the 'probe only' spectrum. Excited state bands are indicated in bold.

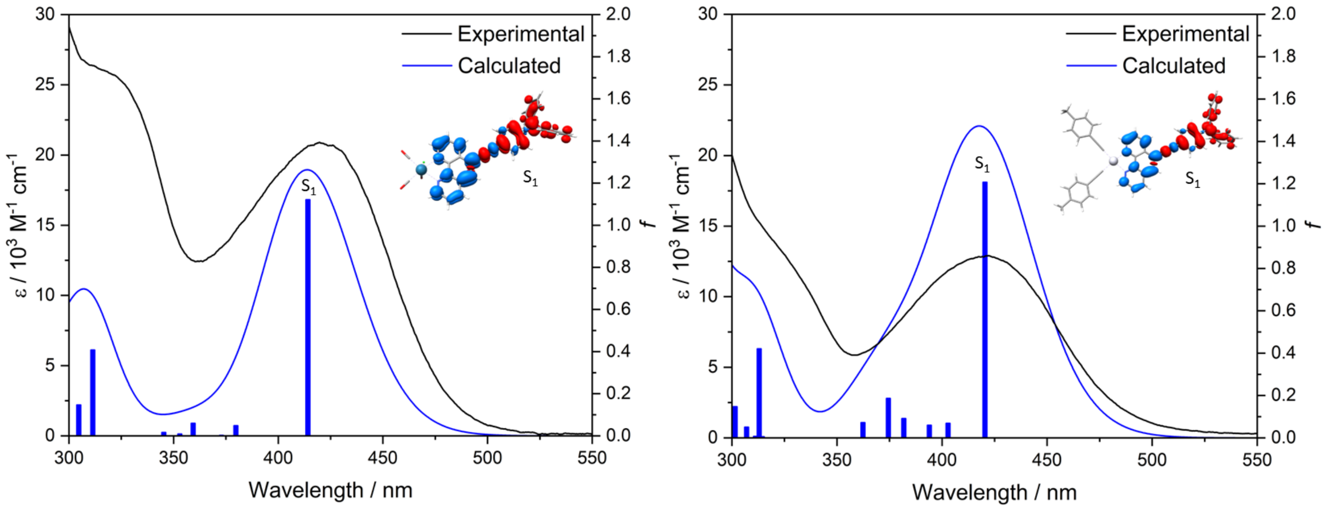

Figure S11. Experimental (black line, CH_2Cl_2) and TD-DFT (B3LYP35, def2-SVP, CH_2Cl_2 solvent field) (blue line) calculated electronic absorption spectra of **Re-CC-TPA** (left) and **Pt-CC-TPA** (right). The charge density difference (CDD) of the S_1 state (ILCT) is shown (red to blue).

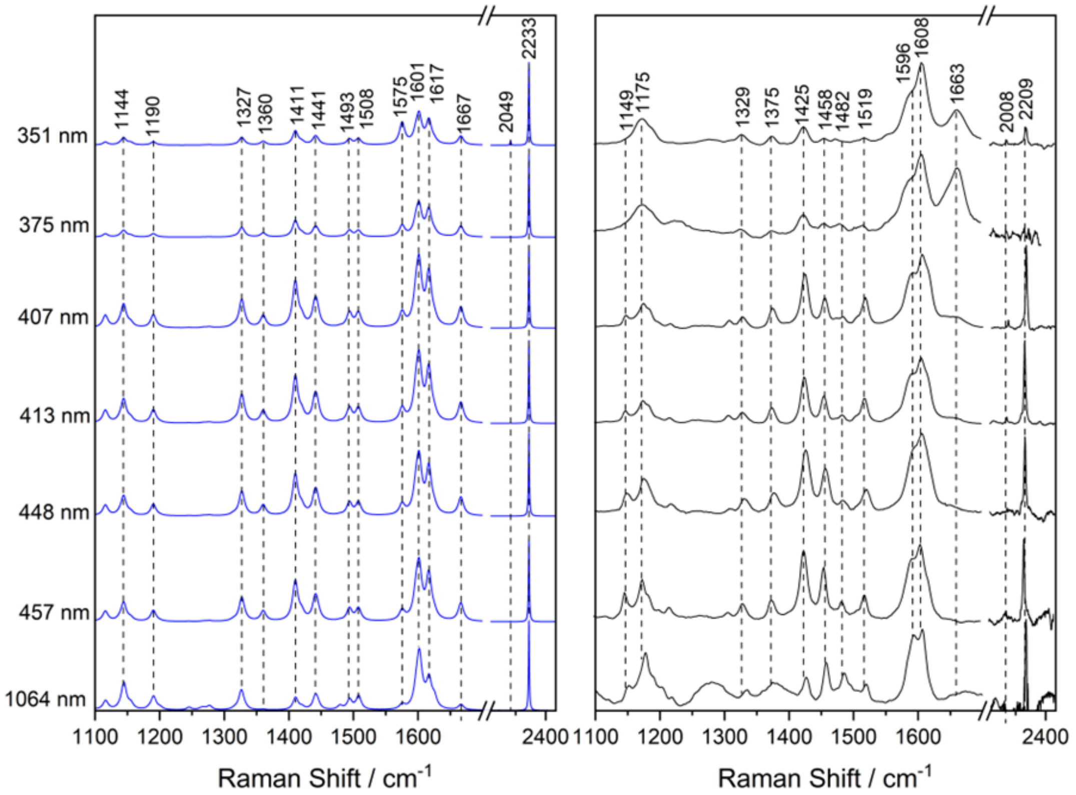

Figure S12. Simulated (left) and experimental (right) resonance Raman spectra of **Re-CC-TPA** obtained in CH_2Cl_2.

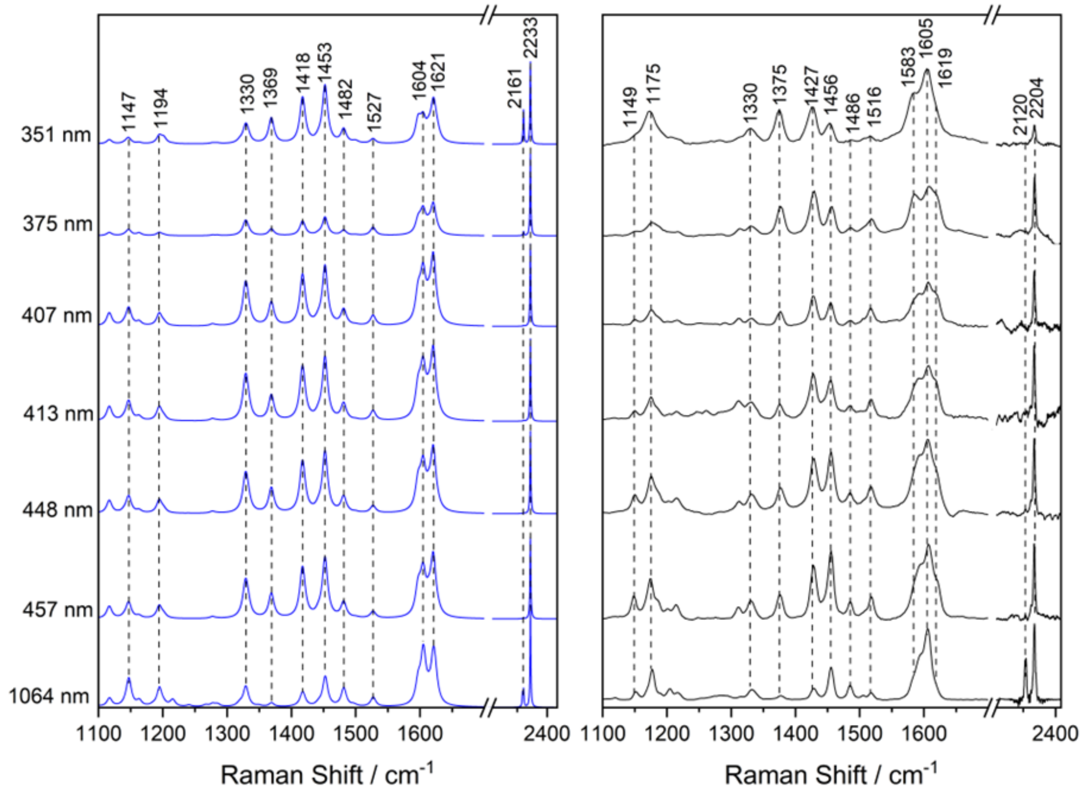

Figure S13. Simulated (left) and experimental (right) resonance Raman spectra of **Pt-CC-TPA** obtained in CH_2Cl_2.

Table S21. TDDFT calculations of **Re-CC-TPA** detailing the lowest eight singlet states obtained from the FC geometry using the def2-SVP basis set. CDDs are given for each transition (red→blue) and the molecular orbitals are shown below in blue and green.

**Re-CC-TPA**	Energy / eV	λ / nm	*f*	Wgt %	From	To	CDD
**S_1**	2.99	414	1.1208	92	154	155	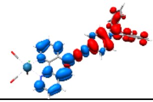
**S_2**	3.26	380	0.0480	86	154	156	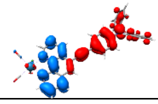
**S_3**	3.33	373	0.0021	19<br>79	153<br>153	155<br>156	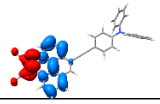
**S_4**	3.45	359	0.0596	21<br>69	152<br>152	155<br>156	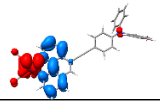
**S_5**	3.51	353	0.0085	77<br>19	153<br>153	155<br>156	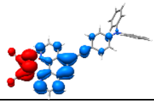
**S_6**	3.59	345	0.0157	74<br>23	152<br>152	155<br>156	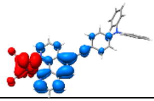
**S_7**	3.91	317	0.0000	15<br>79	150<br>150	155<br>156	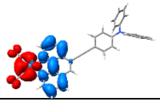
**S_8**	3.98	311	0.4075	83	154	157	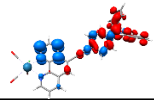

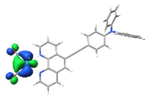

150

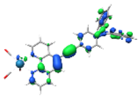

151

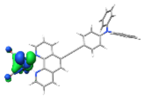

152

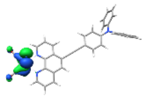

153

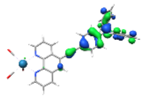

154

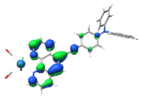

155

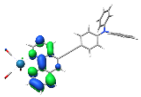

156

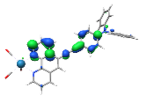

157

Table S22. TDDFT calculations of **Pt-CC-TPA** detailing the lowest eight singlet states obtained from the FC geometry using the def2-SVP basis set. CDDs are given for each transition (red→blue) and the molecular orbitals are shown below in blue and green.

**Pt-CC-TPA**	Energy / eV	λ / nm	*f*	Wgt %	From	To	CDD
**S_1**	2.95	420	1.2072	85	187	188	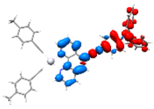
**S_2**	3.08	403	0.0682	49<br>46	186<br>186	188<br>189	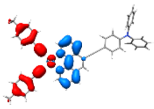
**S_3**	3.15	394	0.0593	10<br>9<br>66	185<br>185<br>187	188<br>189<br>189	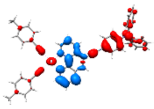
**S_4**	3.25	382	0.0908	11<br>19<br>17<br>25<br>21	185<br>185<br>186<br>186<br>187	188<br>189<br>188<br>189<br>189	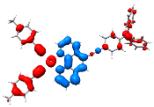
**S_5**	3.31	374	0.1861	34<br>14<br>26<br>20	185<br>185<br>186<br>186	188<br>189<br>188<br>189	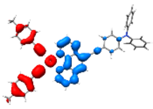
**S_6**	3.42	362	0.0719	39<br>54	185<br>185	188<br>189	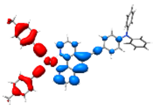
**S_7**	3.71	334	0.0002	44<br>53	184<br>184	188<br>189	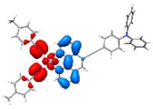
**S_8**	3.94	314	0.0049	43<br>55	181<br>181	188<br>189	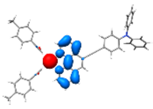

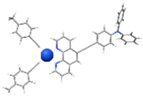<br>181	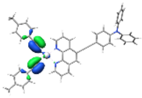<br>182	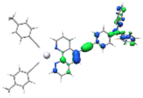<br>183	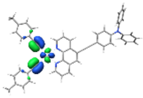<br>184	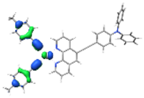<br>185
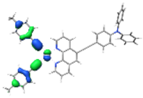<br>186	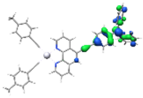<br>187	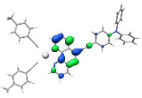<br>188	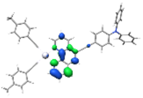<br>189	

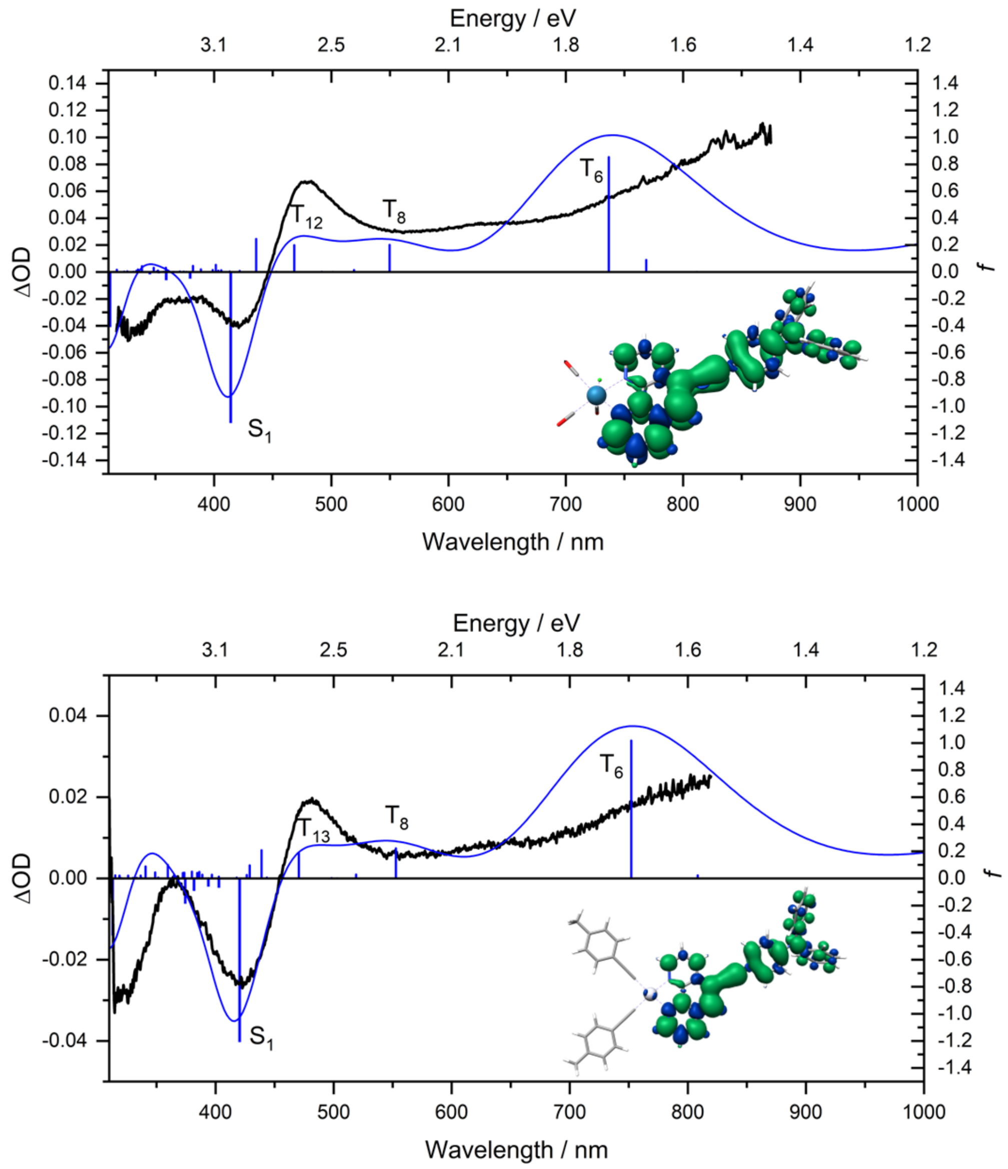

Figure S14. Simulated (blue - B3LYP35, def2-SVP, CH_2Cl_2 solvent field) and experimental (black) transient absorption spectrum of **Re-CC-TPA** (upper) and **Pt-CC-TPA** (lower) obtained in degassed CH_2Cl_2. The downward vertical bars correspond to ground state bleach (singlet-singlet transitions in S_0 equilibrium), while upward vertical bars correspond are associated with excited state absorption (triplet-triplet transitions in T_1 structure). The inset shows the spin density distribution of the complex in its optimised T_1 geometry.

Table S23: Spin orbit couplings between the first four singlet and five triplet states of **Re-CC-TPA** in its optimised FC geometry using the a) ZORA-def2-SVP and b) DKH-def2-SVP basis sets. The respective state energies and CDDs (red →blue) are from the TDDFT ORCA calculations.

Re-CC-TPA:
S_0 geometry

			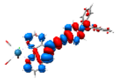	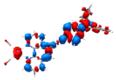	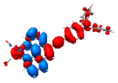	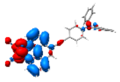	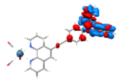
a) ZORA-def2-SVP		E / eV	2.06	2.78	2.96	3.17	3.18
	E / eV		**T_1**	**T_2**	**T_3**	**T_4**	**T_5**
	0.00	**S_0**	5	30	83	237	28
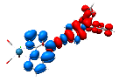	2.99	**S_1**	3	10	11	43	5
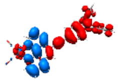	3.26	**S_2**	12	40	27	158	19
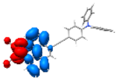	3.28	**S_3**	1	11	236	62	8
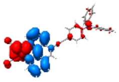	3.42	**S_4**	36	121	31	539	64

Re-CC-TPA:
S_0 geometry

			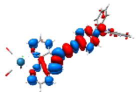	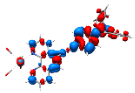	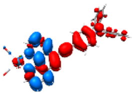	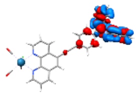	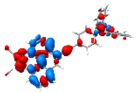
b) DKH-def2-SVP		E / eV	2.06	2.79	3.03	3.18	3.34
	E / eV		**T_1**	**T_2**	**T_3**	**T_4**	**T_5**
	0.00	**S_0**	4	14	50	1	121
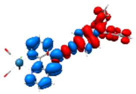	3.02	**S_1**	1	3	4	0	13
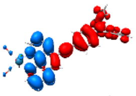	3.32	**S_2**	5	11	3	0	38
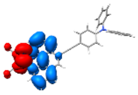	3.58	**S_3**	3	13	145	0	29
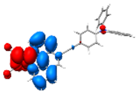	3.66	**S_4**	27	70	23	1	278

Table S24: Spin orbit couplings between the first four singlet and five triplet states of **Pt-CC-TPA** in its optimised FC geometry using the a) ZORA-def2-SVP and b) DKH-def2-SVP basis sets. The respective state energies and CDDs (red →blue) are from the TDDFT ORCA calculations.

Pt-CC-TPA: S_0 geometry

a) ZORA-def2-SVP

			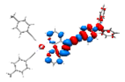	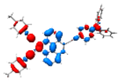	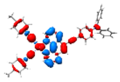	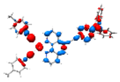	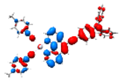
		E / eV	2.05	2.67	2.73	2.82	2.93
	E / eV		**T_1**	**T_2**	**T_3**	**T_4**	**T_5**
	0.00	**S_0**	12	66	8	11	17
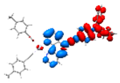	2.93	**S_1**	17	79	18	19	5
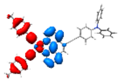	3.03	**S_2**	9	53	408	47	61
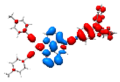	3.11	**S_3**	55	183	3	16	5
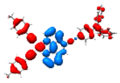	3.22	**S_4**	87	289	56	24	11

Pt-CC-TPA: S_0 geometry

b) DKH-def2-SVP

			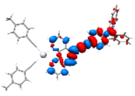	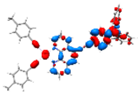	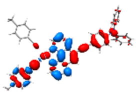	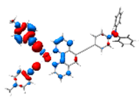	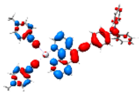
		E / eV	2.06	2.77	2.86	2.88	2.96
	E / eV		**T_1**	**T_2**	**T_3**	**T_4**	**T_5**
	0.00	**S_0**	7	21	10	7	14
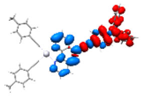	2.97	**S_1**	5	15	7	11	4
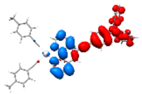	3.19	**S_2**	16	35	3	19	4
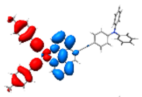	3.29	**S_3**	7	30	198	62	25
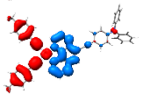	3.42	**S_4**	64	148	16	56	6

Table S25. Rotamers of the **Re-trz-TPA** in both singlet and triplet multiplicities. The rotamers are labelled A-D with respect to increasing energy relative to the lowest identified S_0 state. The measured dihedral angle is indicated in red in the corresponding structure.

Re-trz-TPA				
Label	Dihedral Angle / °		Relative energy / eV	
	S_0	T_1	S_0	T_1
A	126.3	125.9	0.0000	2.3474
B	-128.7	-129.1	0.0117	2.3605
C	56.3	60.9	0.0253	2.3755
D	-53.7	-54.1	0.0369	2.3821

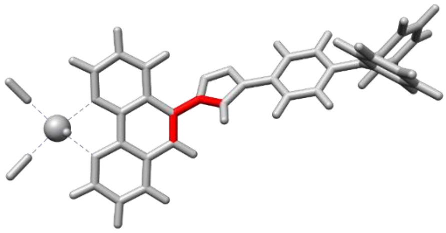

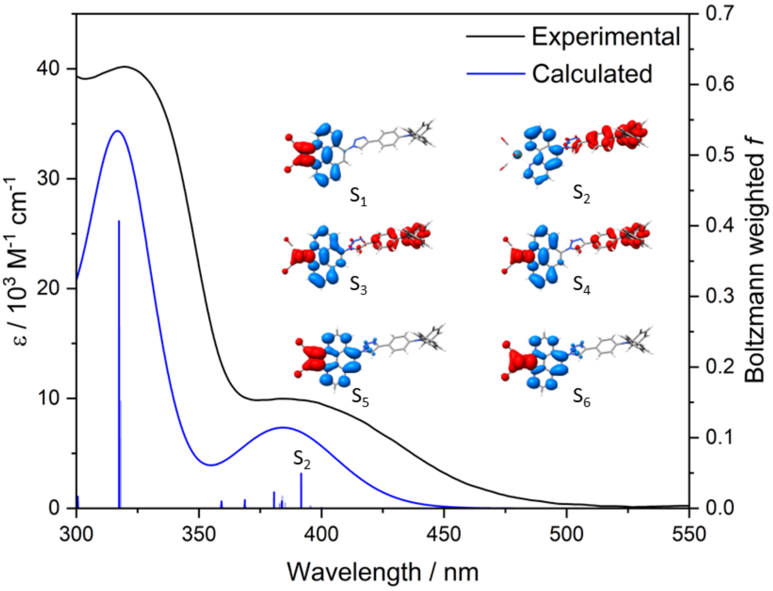

Figure S15. Experimental (black line) and the Boltzmann weighted TD-DFT (B3LYP35, def2-SVP, CH_2Cl_2 solvent field) (blue line) calculated electronic absorption spectra of **Re-trz-TPA**. The transitions shown in bold correspond to those of rotamer A and the oscillator strengths are scaled based on the Boltzmann weighting of the rotamers. The charge density differences (CDDs) of the S_1 – S_6 states of rotamer A are shown.

Table S26. TDDFT calculations of **Re-trz-TPA** (rotamer A) detailing the lowest eight singlet states obtained from the FC geometry using the def2-SVP basis set. CDDs are given for each transition (red→blue) and the molecular orbitals are shown below in blue and green.

**Re-trz-TPA Rotamer A**	Energy / eV	λ / nm	*f*	Wgt %	From	To	CDD
**S_1**	3.10	400	0.0019	94	164	166	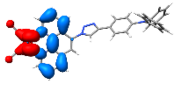
**S_2**	3.17	392	0.1106	49<br>45	165<br>165	166<br>167	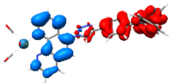
**S_3**	3.23	384	0.0227	41<br>18<br>35	163<br>165<br>165	166<br>166<br>167	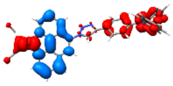
**S_4**	3.26	381	0.0512	47<br>31<br>17	163<br>165<br>165	166<br>166<br>167	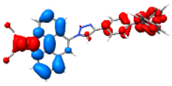
**S_5**	3.36	369	0.0267	90	164	167	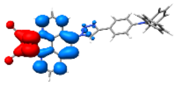
**S_6**	3.45	359	0.0222	94	163	167	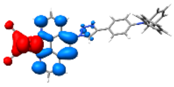
**S_7**	3.69	336	0.0000	92	162	166	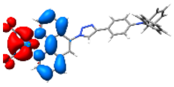
**S_8**	3.91	317	0.9126	27<br>67	165<br>165	168<br>169	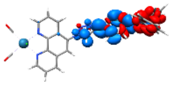

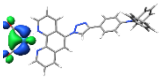
162

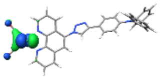
163

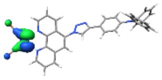
164

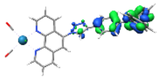
165

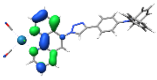
166

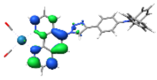
167

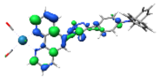
168

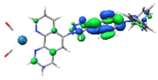
169

Table S27. TDDFT calculations of **Re-trz-TPA** (rotamer B) detailing the lowest eight singlet states obtained from the FC geometry using the def2-SVP basis set. CDDs are given for each transition (red→blue) and the molecular orbitals are shown below in blue and green.

**Re-trz-TPA Rotamer B**	Energy / eV	λ / nm	*f*	Wgt %	From	To	CDD
**S₁**	3.10	400	0.0018	93	164	166	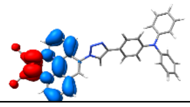
**S₂**	3.17	392	0.1269	47	165	166	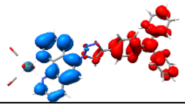
				47	165	167	
**S₃**	3.23	384	0.0235	43	163	166	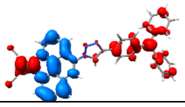
				18	165	166	
				34	165	167	
**S₄**	3.26	380	0.0515	45	163	166	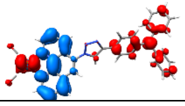
				34	165	166	
				16	165	167	
**S₅**	3.37	368	0.0249	91	164	167	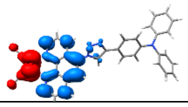
**S₆**	3.45	359	0.0225	94	163	167	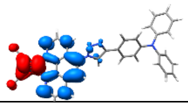
**S₇**	3.69	336	0.0006	91	162	166	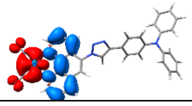
**S₈**	3.90	318	0.9121	26	165	168	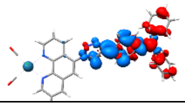
				68	165	169	

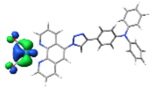
162

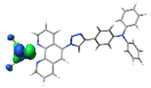
163

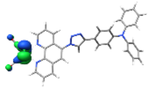
164

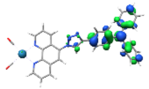
165

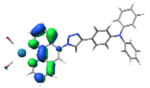
166

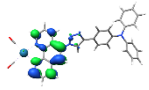
167

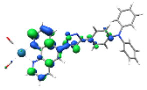
168

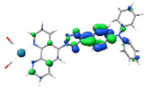
169

Table S28. TDDFT calculations of **Re-trz-TPA** (rotamer C) detailing the lowest eight singlet states obtained from the FC geometry using the def2-SVP basis set. CDDs are given for each transition (red→blue) and the molecular orbitals are shown below in blue and green.

**Re-trz-TPA Rotamer C**	Energy / eV	λ / nm	*f*	Wgt %	From	To	CDD
**S_1**	3.08	402	0.0019	93	164	166	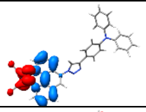
**S_2**	3.14	395	0.0190	78<br>20	165<br>165	166<br>167	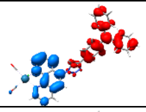
**S_3**	3.23	384	0.1046	90	163	166	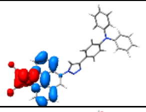
**S_4**	3.24	383	0.0387	20<br>77	165<br>165	166<br>167	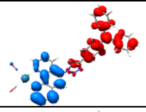
**S_5**	3.37	368	0.0246	91	164	167	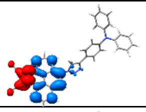
**S_6**	3.46	359	0.0258	94	163	167	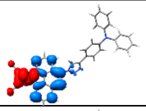
**S_7**	3.67	338	0.0007	91	162	166	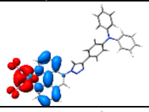
**S_8**	3.90	318	0.9123	20<br>73	165<br>165	168<br>169	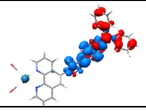

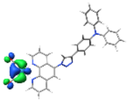
162

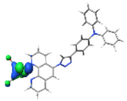
163

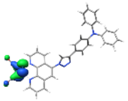
164

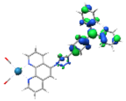
165

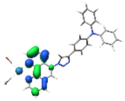
166

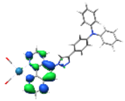
167

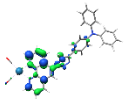
168

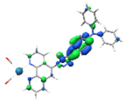
169

Table S29. TDDFT calculations of **Re-trz-TPA** (rotamer D) detailing the lowest eight singlet states obtained from the FC geometry using the def2-SVP basis set. CDDs are given for each transition (red→blue) and the molecular orbitals are shown below in blue and green.

**Re-trz-TPA Rotamer D**	Energy / eV	λ / nm	*f*	Wgt %	From	To	CDD
**S_1**	3.08	403	0.0019	93	164	166	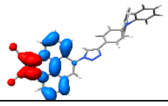
**S_2**	3.13	396	0.0284	77<br>20	165<br>165	166<br>167	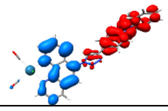
**S_3**	3.22	385	0.0739	89	163	166	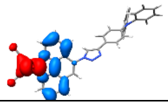
**S_4**	3.24	383	0.0589	22<br>75	165<br>165	166<br>167	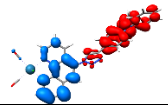
**S_5**	3.36	369	0.0245	90	164	167	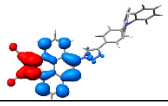
**S_6**	3.45	360	0.0230	94	163	167	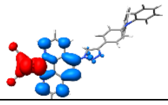
**S_7**	3.67	338	0.0004	91	162	166	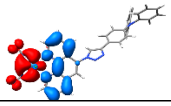
**S_8**	3.90	318	0.9400	22<br>71	165<br>165	168<br>169	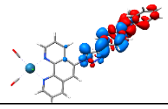

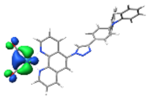

162

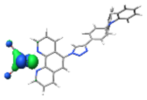

163

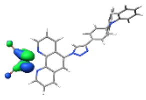

164

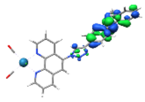

165

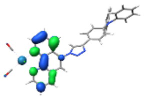

166

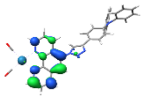

167

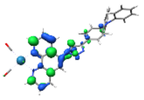

168

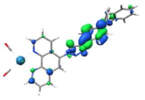

169

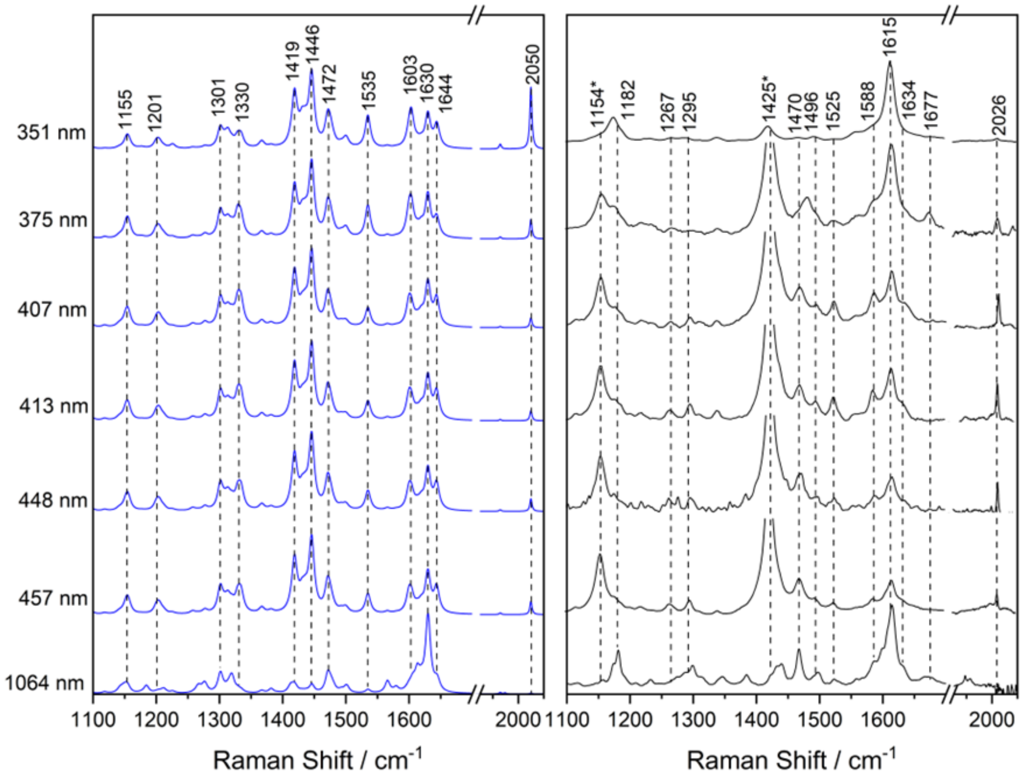

Figure S16. Boltzmann weighted simulated (left) and experimental (right) resonance Raman spectra of **Re-trz-TPA** obtained in CH_2Cl_2.

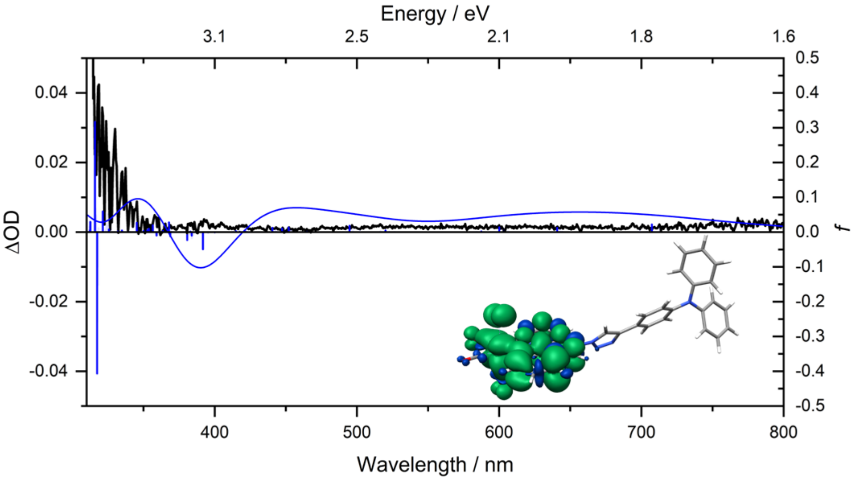

Figure S17. Boltzmann weighted simulated (blue - B3LYP35, def2-SVP, CH_2Cl_2 solvent field) and experimental (black) transient absorption spectra of **Re-trz-TPA** obtained in degassed CH_2Cl_2. The downward vertical bars correspond to ground state bleach (singlet-singlet transitions in S_0 equilibrium), while upward vertical bars correspond are associated with excited state absorption (triplet-triplet transitions in T_1 structure). The oscillator strengths have been scaled based on the Boltzmann weighting. The inset shows the spin density distribution of the complex in the optimised T_1 geometry which would likely be obtained from excitation of the S_0 rotamer A.

Table S30: Spin orbit couplings between the first five singlet and five triplet states of **Re-trz-TPA** in its lowest optimised FC geometry (Rotamer A) using the a) ZORA-def2-SVP and b) DKH-def2-SVP basis sets. The respective state energies and CDDs (red →blue) are from the TDDFT ORCA calculations.

Re-trz-TPA:
S_0 Rotamer A
a) ZORA-def2-SVP

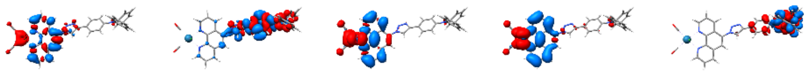

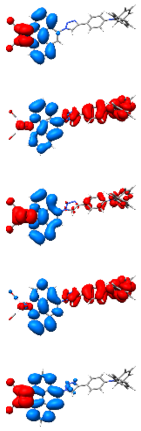

	E / eV	2.53	2.77	2.94	3.00	3.13
E / eV		**T_1**	**T_2**	**T_3**	**T_4**	**T_5**
0.00	**S_0**	44	13	178	205	1
3.05	**S_1**	5	3	585	16	1
3.17	**S_2**	72	18	7	211	1
3.20	**S_3**	191	45	8	509	2
3.25	**S_4**	54	14	10	162	1
3.32	**S_5**	102	10	19	210	2

Re-trz-TPA:
S_0 Rotamer A
b) DKH-def2-SVP

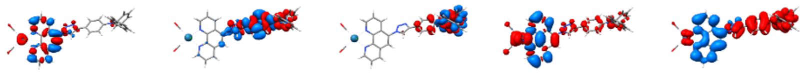

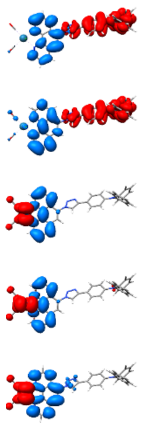

	E / eV	2.57	2.78	3.13	3.16	3.20
E / eV		**T_1**	**T_2**	**T_3**	**T_4**	**T_5**
0.00	**S_0**	25	5	2	163	98
3.22	**S_1**	5	1	0	5	12
3.31	**S_2**	14	3	0	27	26
3.36	**S_3**	7	0	1	480	3
3.47	**S_4**	134	27	2	19	278
3.60	**S_5**	106	16	0	25	10

Section 2: Experimental

Caution! Chemicals should always be handled with care, some of the reagents and solvents used herein are flammable and/or toxic and risk assessments should be carried out before attempting any of the reported procedures. Appropriate personal protective equipment should be used at all times.

Synthesis of the ligands

General

Sodium (4-hexylthiophen-2-yl)boronate,[1] 5-Azido-1,10-phenanthroline,[2] 5-{4-[N,N-(4-cyanophenyl)aminophenyl]}-1,10-phenanthroline (TPA-CN) and 5-{4-[N,N-(4-methoxyphenyl)aminophenyl]}-1,10-phenanthroline (TPA-OMe)[3] were synthesized according to previously published procedures. Other chemicals were obtained commercially and were used without further purification unless stated differently.

Synthesis of thio-TPA:

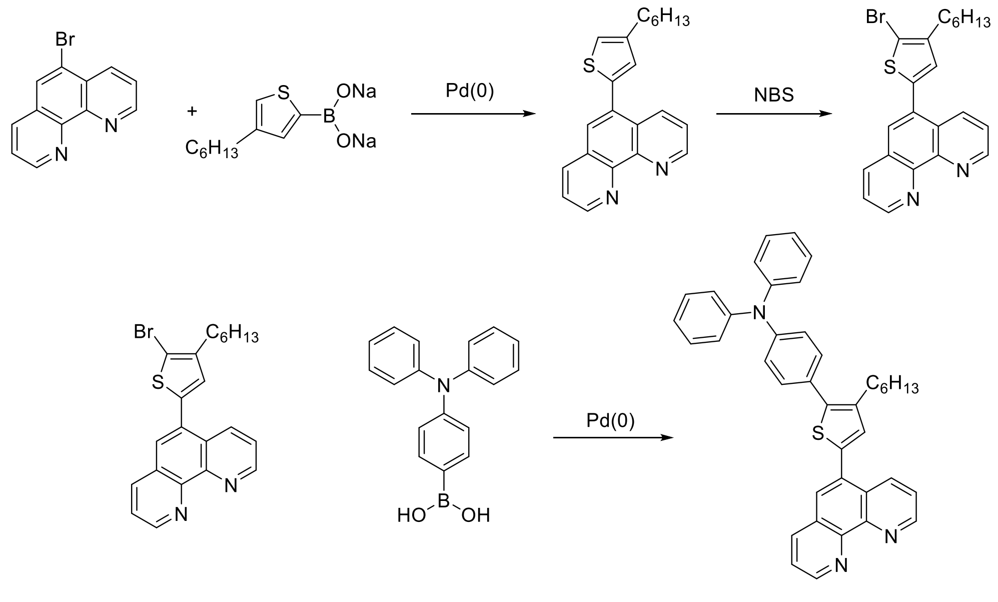

5-(4-Hexylthiophen-2-yl)-1,10-phenanthroline:

5-Bromo-1,10-phenanthroline hydrate (1.108 g, 4 mmol) and sodium (4-hexylthiophen-2-yl)boronate (1.537 g, 6 mmol) were dissolved in dimethoxyethane (35 mL) then $Pd(PPh_3)_4$ (0.421 g, 0.4 mmol) was added followed by aqueous solution of potassium carbonate (15 mL, 1M). The resulting mixture was stirred at 90°C overnight. After cooling, the mixture was extracted with chloroform. The organic layer was separated, dried over magnesium sulfate and evaporated to dryness. The resulting oil was purified on silica using 3% methanol in dichloromethane as an eluent to give slightly yellow oil.

Yield: 62 %, ^{1}H NMR (500 MHz, $CDCl_3$, TMS): 9.25 – 9.17 (m, 2H), 8.65 (dd, 1H, J = 1.7 and 8.4 Hz), 8.24 (dd, 1H, J = 1.7 and 8.4 Hz), 7.88 (s, 1H), 7.67 – 7.61 (m, 2H), 7.13 (d, 1H, J = 1.5 Hz), 7.08 (d, 1H, J = 1.5 Hz), 2.72 (t, 2H, J = 7.7 Hz), 1.77 – 1.66 (m, 2H), 1.47 – 1.31 (m, 6H), 0.91 (t, 3H, 7.1 Hz); HRMS (ESI, $(M+1)^+$): found:347.1582; requires for $C_{22}H_{23}N_2S$: 347.1776.

5-(5-Bromo-4-hexylthiophen-2-yl)-1,10-phenanthroline:

5-(4-Hexylthiophen-2-yl)-1,10-phenanthroline (1.063 g, 2.5 mmol) was dissolved in tetrahydrofuran (30 mL), cooled to 0°C and NBS (0.530 g, 3.0 mmol) was added by portions. The resulting mixture was stirred at 0°C for 30 min then overnight at room temp. Afterwards, the mixture was washed by saturated aqueous solution of sodium thiosulfate then a couple times with water to give beige solid.

Yield: 99 %, ^{1}H NMR (500 MHz, $CDCl_3$, TMS): 9.23 (dd, 1H, J = 1.7 and 4.4 Hz), 9.21 (dd, 1H, J = 1.7 and 4.4 Hz), 8.61 (dd, 1H, J = 1.7 and 8.4 Hz), 8.25 (dd, 1H. J = 1.7 and 8.2 Hz), 7.85 (s, 1H), 7.68 – 7.63 (m, 2H), 7.00 (s, 1H), 2.67 (t, 2H, J = 7.7 Hz), 1.73 – 1.64 (m, 2H), 1.47 – 1.31 (m, 6H), 0.91 (t, 3H, J = 7.1 Hz); HRMS (ESI, $(M+1)^+$): found:425.0692; requires for $C_{22}H_{23}N_2SBr$: 425.0682.

5-[5-(4-Diphenylaminophenylene)-4-hexylthiophen-2-yl]-1,10-phenanthroline:

5-(5-Bromo-4-hexylthiophen-2-yl)-1,10-phenanthroline (1.060 g, 2.5 mmol) and [4-(diphenylamino)phenyl]boronic acid (1.086 g, 3.8 mmol) were dissolved in dimethoxyethane (40 mL) then $Pd(PPh_3)_4$ (0.263 g, 0.25 mmol) was added followed by aqueous solution of potassium carbonate (20 mL, 1M). The resulting mixture was stirred at 90°C overnight. After cooling, the mixture was extracted with chloroform. The organic layer was separated, dried over magnesium sulfate and evaporated to dryness. The resulting oil was purified on silica using 3% methanol in dichloromethane as an eluent to give slightly yellow solid.

Yield: 55 %, ^{1}H NMR (500 MHz, $CDCl_3$, TMS): 9.23 (dd, 1H, J = 1.7 and 4.2 Hz), 9.19 (dd, 1H, J = 1.7 and 4.2 Hz), 8.78 (dd, 1H, J = 1.7 and 8.4 Hz), 8.25 (dd, 1H, J = 1.7 and 8.4 Hz), 7.92 (s, 1H), 7.71 – 7.03 (m, 17H), 2.77 (t, 2H, 7.8 Hz), 1.76 – 1.66 (m, 2H), 1.45 – 1.35 (m, 2H), 1.33 – 1.27 (m, 4H), 0.89 (t, 3H, J = 6.9 Hz); HRMS (ESI, $(M+1)^+$): found:590.2614; requires for $C_{40}H_{36}N_3S$: 590.2624.

Synthesis of trz-TPA:

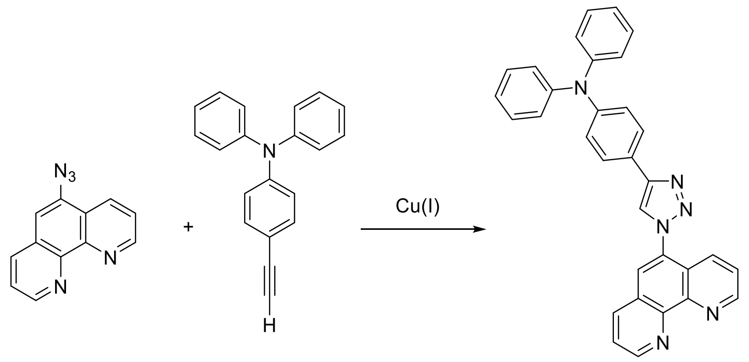

5-{4-[4-(Diphenylamino)phenyl]-1,2,3-triazol-1-yl}-1,10-phenanthroline:

5-Azido-1,10-phenanthroline (0.630 g, 2.9 mmol) and 4-ethynyl-*N,N*-diphenylaniline (0.767 g, 2.9 mmol) were dissolved in dimethylformamide (40 mL). Copper(II) sulfate petahydrate was dissolved in water (20 mL) and two solutions were mixed. To the resulting solution sodium ascorbate (1.694 g, 8.6 mmol) was added and the resulting mixture was stirred at 65ºC for 24 h then poured into saturated aqueous solution of EDTA (1 L). Dichloromethane (150 mL) was added, and two-phased mixture was vigorously stirred overnight. The organic phase was separated, dried over magnesium sulfate and evaporated to dryness. The remaining was purified on silica using 10% methanol in dichloromethane as an eluent to give white solid.

Yield: 78 %, ^{1}H NMR (500 MHz, $CDCl_3$, TMS): 9.32 (dd, 1H, J = 1.7 and 4.4 Hz), 8.22 (dd, 1H, J = 1.7 and 4.4 Hz), 8.16 (s, 1H), 8.03 (s, 1H), 7.86 – 7.81 (m, 2H), 7.75 (dd, 1H, J = 4.4 and 8.0 Hz), 7.70 (dd, 1H, J = 4.4 and 8.0 Hz), 7.33 – 7.26 (m, 4H), 7.22 – 7.13 (m, 6H), 7.10 – 7.04 (m, 2H); HRMS (ESI, $(M+1)^+$): found:491.1988; requires for $C_{32}H_{23}N_6$: 491.1979.

Synthesis of CC-TPA:

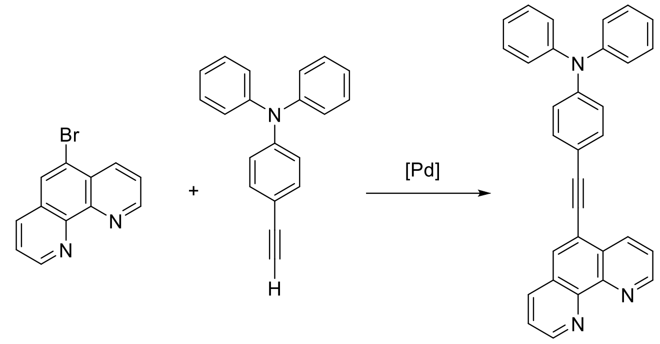

5-[(4-Diphenylamino)phenylethynyl]-1,10-phenanthroline:

5-Bromo-1,10-phenanthroline (0.777 g, 3.0 mmol), 4-ethynyl-*N,N*-diphenylaniline (1.212 g, 4.5 mmol), Bis(triphenylphosphino)palladium(II) chloride (0.201 g, 0.3 mmol), and copper(I) iodide (0.054 g, 0.6 mmol) were suspended in a mixture of tetrahydrofuran (15 mL and dry triethylamine (15 mL). The resulting suspension was thaw-degassed (3 times) and refluxed for 20 h under argon. Afterwards, the solvents were removed under vacuum. The resulting oil was dissolved in dichloromethane and adsorbed on a pad of silica. The silica was washed with dichloromethane till eluent was colourless – this fraction was discarded. Then the silica pad was washed with 10% methanol in dichloromethane. The eluent was added to aqueous saturated solution of EDTA (600 mL) and vigorously stirred overnight. The organic phase was separated, dried over magnesium sulfate and evaporated to dryness. The remaining was purified on silica using 10% methanol in dichloromethane as an eluent to give deep-yellow viscous oil.

Yield: 83 %, ^{1}H NMR (500 MHz, $CDCl_3$, TMS): 9.26 – 9.13 (m, 2H), 8.82 (dd, 1H, J = 1.8 and 8.3 Hz), 8.21 (dd, 1H, J = 1.8 and 8.3 Hz), 8.06 (s, 1H), 7.73 (dd, 1H, J = 4.4 and 8.3 Hz), 7.64 (dd, 1H, J = 4.4 and 8.3 Hz), 7.53 – 7.48 (m, 2H), 7.34 – 7.28 (m, 4H), 7.18 – 7.04 (m, 8H); HRMS (ESI, $(M+1)^+$): found:448.1820; requires for $C_{32}H_{22}N_3$: 448.1808.

Synthesis of the complexes

General

Unless otherwise stated, all reagents were purchased from commercial sources and used without further purification. The Pt(II) starting material, [Pt(COD)(4-pentylphenylacetylide)$_2$] was synthesised as previously reported.[3] Solvents were laboratory reagent grade. Petroleum ether refers to the fraction of petrol boiling in the range 40-60 °C. ^{1}H and ^{13}C NMR spectra were recorded on either a 400 MHz Varian 400-MR or Varian 500 MHz AR spectrometer. Chemical shifts are reported in parts per million and referenced to residual solvent peaks ($CDCl_3$: ^{1}H δ 7.26 ppm, ^{13}C δ 77.16 ppm; CD_3CN: ^{1}H δ 1.94, ^{13}C δ 1.32, 118.26 ppm, d_6-DMSO: ^{1}H δ 2.50 ppm; ^{13}C δ 39.52 ppm, d_7-DMF: ^{1}H δ 8.03 ppm; ^{13}C δ 163.15 ppm). Coupling constants (J) are reported in Hertz (Hz). Standard abbreviations indicating multiplicity were used as follows: m = multiplet, q = quartet, quin =quintet = triplet, dt = double triplet, d = doublet, dd = double doublet, s = singlet, br = broad. IR spectra were recorded on a Bruker ALPHA FT-IR spectrometer with an attached ALPHA-P measurement module. Microanalyses were performed at the Campbell Microanalytical Laboratory at the University of Otago. Electrospray mass spectra (ESMS) were collected on a Bruker micrOTOF-Q spectrometer.

Note: The two acetylide ligands on each Pt(II) phen complex are in slightly different NMR environments. These are overlapping in the ^{1}H NMR spectra, and are characterised as a single resonance in each case. They can be distinguished in some cases in the ^{13}C NMR spectra. Coincident peaks in the ^{13}C NMR spectra are noted in the experimental details with a # symbol.

Synthesis of Re-thio-TPA

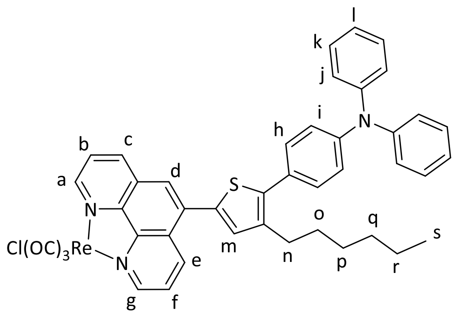

A suspension of **thio-TPA** (100 mg, 0.17 mmol) and [Re(CO)$_5$Cl] (58 mg, 0.16 mmol) in toluene (25 mL) was heated overnight at 80 °C. The solvent was removed under vacuum to afford the product as an orange solid (142 mg, 0.16 mmol, 98%). ^{1}H NMR (400 MHz, CDCl$_3$, 298 K) δ: 9.43 (1H, d, J = 4.5 Hz, H$_g$), 9.37 (1H, d, J = 4.3 Hz, H$_a$), 9.05 (1H, d, J = 8.2 Hz, H$_e$), 8.52 (1H, d, J = 8.0 Hz, H$_c$), 8.09 (1H, s, H$_d$), 7.91 – 7.85 (2H, m, H$_{b,f}$), 7.36 (2H, d, J = 8.5 Hz, H$_h$), 7.30 (4H, t, J = 8.0 Hz, H$_k$), 7.19 (1H, s, H$_m$), 7.18 (4H, d, J = 7.8 Hz, H$_j$), 7.13 (2H, d, J = 8.6 Hz, H$_i$), 7.08 (1H, t, J = 7.3 Hz, H$_l$), 2.77 (2H, t, J = 7.9 Hz, H$_n$), 1.70 (2H, quin, J = 7.6 Hz, H$_o$), 1.39 (2H, quin, J = 8.6 Hz, H$_p$), 1.32 – 1.26 (4H, m, H$_{q,r}$), 0.89 (3H, t, J = 7.8 Hz, H$_s$). ^{13}C NMR (100 MHz, CDCl$_3$, 298 K) δ: 197.2, 197.1, 189.5, 153.1 (C$_g$), 152.9 (C$_a$), 147.9, 147.6, 147.5, 146.5, 141.2, 139.6, 138.0 (C$_c$), 137.2 (C$_e$), 134.4, 133.9, 131.8 (C$_m$), 130.5, 130.3, 130.1 (C$_h$), 129.6 (C$_k$), 127.6, 127.1, 126.2, 125.9, 125.0 (C$_j$), 123.6 (C$_l$), 122.9 (C$_i$), 31.8 31.2 (C$_o$), 29.3 (C$_p$), 29.0 (C$_n$), 22.8, 14.2 (C$_s$). HR ESI-MS (CDCl$_3$/MeOH) m/z = 860.1932 [M – Cl]$^+$ (calc. for $C_{43}H_{35}N_3O_3ReS$, 860.1952), m/z = 878.2049 [M – Cl + H_2O]$^+$ (calc. for $C_{43}H_{37}N_3O_7Re$, 878.2057). Anal. calcd. for $C_{43}H_{35}ClN_3O_3ReS$·1.25Toluene: C, 61.50; H, 4.49; N, 4.16%. Found: C, 61.26; H, 4.82; N, 4.41%.

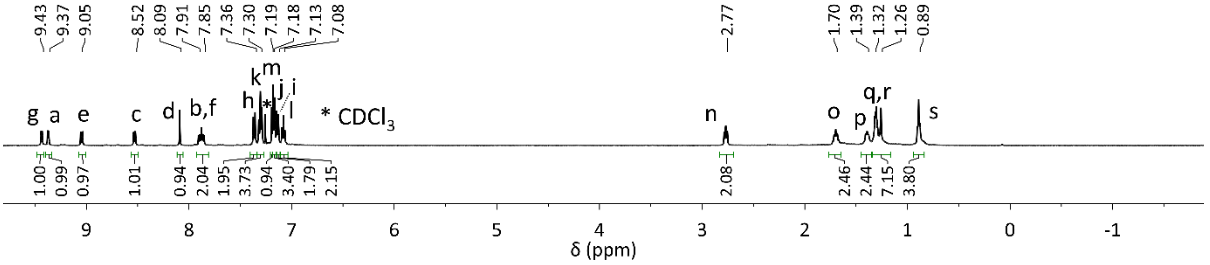

Figure S18: ^{1}H NMR (400 MHz, CDCl$_3$, 298 K) of **Re-thio-TPA**.

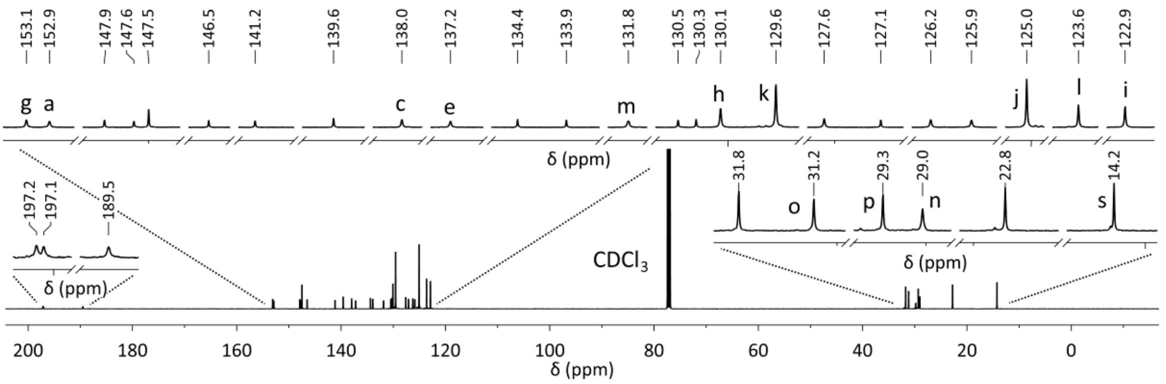

Figure S19: ^{13}C NMR (100 MHz, CDCl$_3$, 298 K) of **Re-thio-TPA**.

Synthesis of Re-CC-TPA

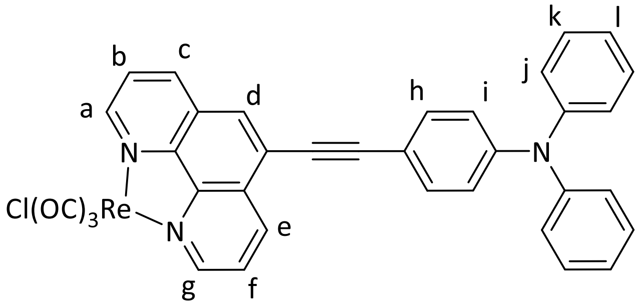

A solution of **CC-TPA** (104 mg, 0.239 mmol) and [Re(CO)$_5$Cl] (84 mg, 0.24 mmol) in toluene (25 mL) was heated overnight at 80 °C. After removal of the solvent under vacuum, the residue was dissolved in minimal dichloromethane. Addition of pet ether, brought about precipitation and the precipitated product was collected by filtration and washed with pet ether to afford the product as a yellow solid (140 mg, 0.191 mmol, 80%). ^{1}H NMR (100 MHz, CDCl$_3$, 298 K) δ: 9.42 (1H, d, J = 5.1 Hz, H$_g$), 9.33 (1H, d, J = 5.1 Hz, H$_a$), 9.02 (1H, d, J = 8.3 Hz, H$_e$), 8.46 (1H, d, J = 8.1 Hz, H$_c$), 8.16 (1H, s, H$_d$), 7.93 (1H, dd, J = 8.4, 5.1 Hz, H$_f$), 7.83 (1H, dd, J = 8.4, 5.1 Hz, H$_b$), 7.51 (2H, d, J = 8.7 Hz, H$_h$), 7.33 (4H, t, J = 8.1 Hz, H$_k$), 7.18 – 7.11 (6H, m, H$_{j,l}$), 7.08 (2H, d, J = 7.7 Hz, H$_i$). ^{13}C NMR (100 MHz, CDCl$_3$, 298 K) δ: 197.1, 197.0, 189.4, 153.4 (C$_g$), 153.0 (C$_a$), 149.5, 147.1, 146.9, 146.3, 137.6 (C$_e$), 137.3 (C$_c$), 133.2 (C$_h$), 130.9, 130.5, 129.9 (C$_d$), 129.7 (C$_k$), 126.2 (C$_f$), 126.1 (C$_b$), 125.7 (C$_j$), 124.4 (C$_l$), 122.8, 121.5 (C$_i$), 113.5, 99.5, 83.4. HR ESI-MS (CDCl$_3$/MeOH) m/z = 718.1116 [M - Cl]$^+$ (calc. for $C_{35}H_{21}N_3O_3Re$, 718.1135). Anal. calcd. for $C_{35}H_{21}ClN_3O_3Re$: C, 54.51; H, 3.01; N, 5.45%. Found: C, 54.18; H, 2.62; N, 5.13%.

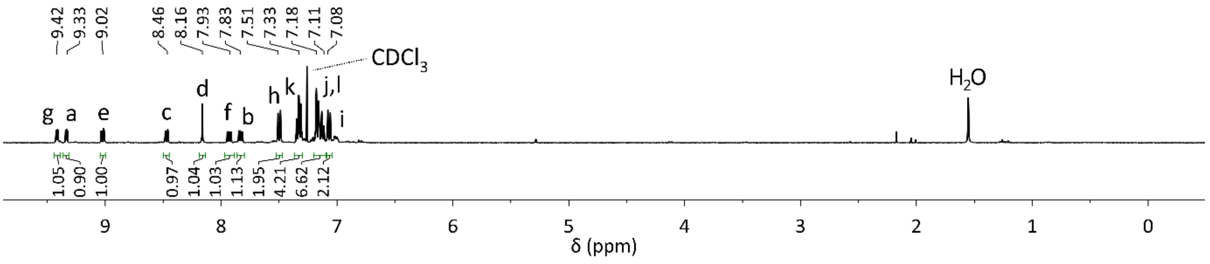

Figure S20: ^{1}H NMR (400 MHz, CDCl$_3$, 298 K) of **Re-CC-TPA**.

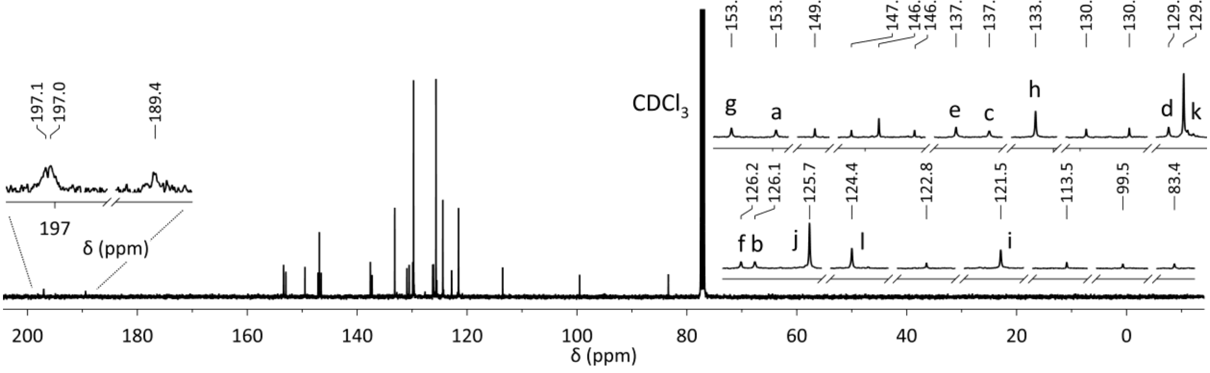

Figure S21: ^{13}C NMR (100 MHz, CDCl$_3$, 298 K) of **Re-CC-TPA**.

Synthesis of Re-trz-TPA

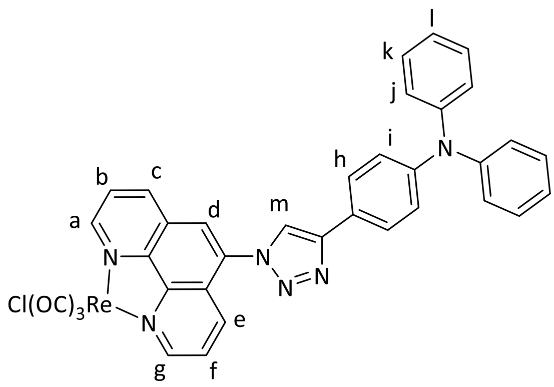

A solution of **trz-TPA** (113 mg, 0.231 mmol) and [Re(CO)$_5$Cl] (81 mg, 0.23 mmol) in toluene (25 mL) was heated overnight at 80 °C. The resultant suspension was collected by filtration and washed with diethyl ether to afford the product as a yellow solid (159 mg, 0.200 mmol, 87%). ^{1}H NMR (500 MHz, d_6-DMSO, 298 K) δ: 9.58 (1H, d, J = 5.0 Hz, H$_g$), 9.56 (1H, d, J = 5.2 Hz, H$_a$), 9.26 (1H, s, H$_m$), 9.07 (1H, d, J = 8.3 Hz, H$_c$), 8.82 (1H, s, H$_d$), 8.76 (1H, d, J = 8.6 Hz, H$_e$), 8.22 (1H, dd, J = 8.3, 5.2 Hz, H$_b$), 8.18 (1H, dd, J = 8.6, 5.1 Hz, H$_f$), 7.92 (2H, d, J = 8.7 Hz, H$_h$), 7.35 (4H, t, J = 8.0 Hz, H$_k$), 7.13 – 7.08 (8H, m, H$_{i,j,l}$). ^{13}C NMR (125 MHz, d_6-DMSO, 298 K) δ: 197.4 (two coincident carbons), 189.7, 154.8, 154.6, 147.5, 147.0, 146.9, 146.4, 145.9, 140.1 (C$_c$), 135.6 (C$_e$), 132.4, 129.7 (C$_k$), 129.0, 127.4, 127.4, 126.8 (C$_h$), 126.2 124.5 (C$_d$), 124.3 (C$_j$), 123.8 (C$_m$), 123.7, 123.4, 123.1. HR ESI-MS (DMSO/acetonitrile) m/z = 819.0859 [M + Na]$^+$ (calc. for $C_{35}H_{22}ClN_6NaO_3Re$, 819.0884). Anal. calcd. for $C_{35}H_{22}ClN_6O_3Re$: C, 52.80; H, 2.78; N, 10.55%. Found: C, 53.09; H, 2.82; N, 10.56%.

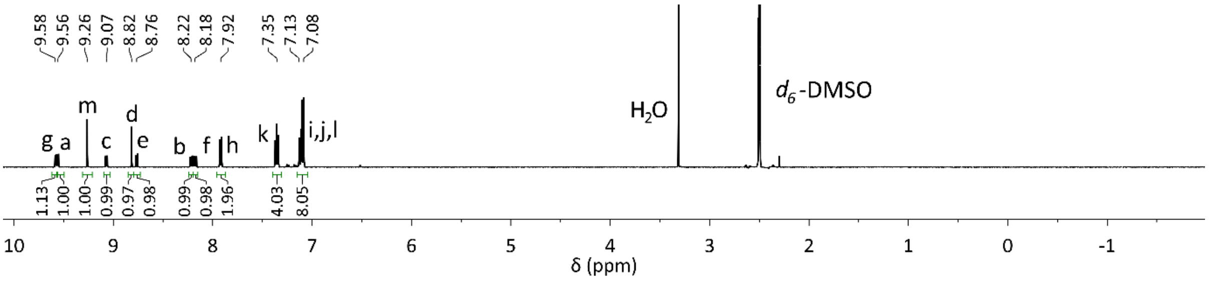

Figure S22: ^{1}H NMR (500 MHz, d_6-DMSO, 298 K) of **Re-trz-TPA**.

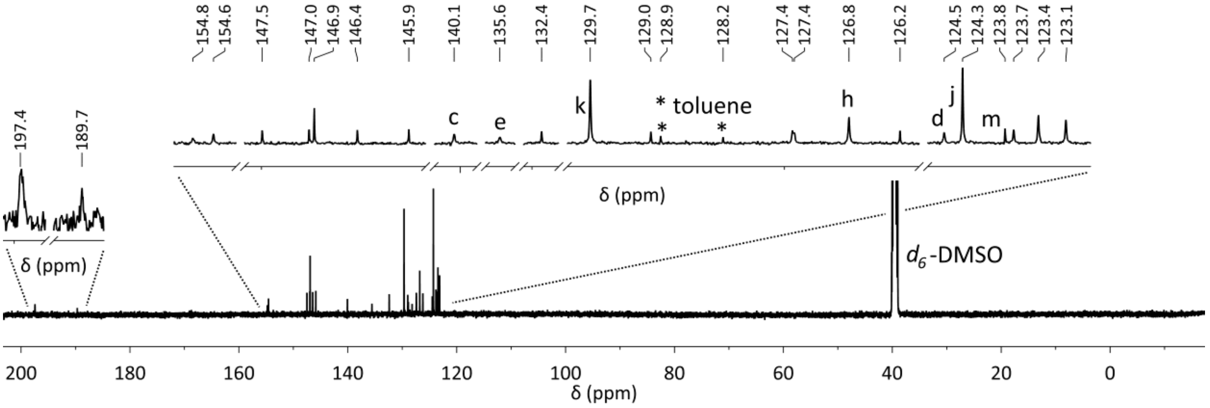

Figure S23: ^{13}C NMR (125 MHz, d_6-DMSO, 298 K) of **Re-trz-TPA**.

Synthesis of Pt-thio-TPA

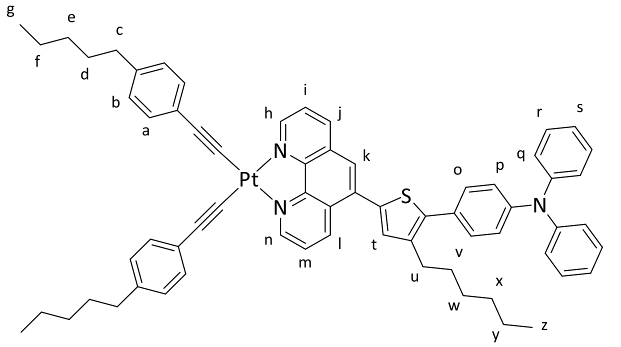

A solution of **thio-TPA** (110 mg, 0.187 mmol) and [Pt(COD)(4-pentylphenylacetylide)$_2$][3] (120 mg, 0.187 mmol) in a 1:5 CH_3CN/dichloromethanesolvent mixture (60 mL) was degassed with N_2 and heated at 45 °C under a nitrogen atmosphere for 3 days. After the removal of the solvent under vacuum, column chromatography on silica (1:1 hexane/dichloromethane, dichloromethane, 1:19 acetone/dichloromethane) gave the product as a red solid (175 mg, 0.155 mmol, 83%). ^{1}H NMR (500 MHz, $CDCl_3$, 298 K) *δ*: 9.94 (1H, d, *J* = 4.7 Hz, H_n), 9.84 (1H, d, *J* = 5.0 Hz, H_h), 9.07 (1H, d, *J* = 8.0 Hz, H_l), 8.56 (1H, d, *J* = 8.1 Hz, H_j), 8.56 (1H, s, H_k), 7.87 – 7.81 (2H, m, $H_{i,m}$), 7.44 (4H, d, *J* = 7.9 Hz, H_a), 7.37 – 7.29 (7H, m, $H_{o,r,t}$), 7.18 (4H, d, *J* = 8.2 Hz, H_q), 7.13 (2H, d, *J* = 8.1 Hz, H_p), 7.09 – 7.06 (6H, m, $H_{b,s}$), 2.73 (2H, t, *J* = 7.8 Hz, H_u), 2.57 (4H, t, *J* = 7.5 Hz, H_c), 1.70 – 1.58 (6H, m, $H_{d,v}$), 1.38 – 1.26 (10H, m, $H_{e,f,w,x,y}$), 0.91 – 0.87 (9H, m, $H_{g,z}$). ^{13}C NMR (125 MHz, $CDCl_3$, 298 K, # = two coincident peaks) *δ*: 151.3, 151.3, 148.3, 149.9, 147.5, 147.1, 140.9, 140.3 (#), 139.7, 137.4, 136.6, 134.6, 132.1 (#), 132.0, 130.1, 129.9 (#), 129.6, 127.9 (#), 127.4, 127.2, 126.4, 126.0, 125.6 (#), 125.0, 123.6, 122.9, 102.4, 102.4, 84.6, 83.9, 36.0 (#), 31.8, 31.7 (#), 31.3 (#), 31.2, 29.4, 29.1, 22.8, 22.7 (#), 14.2, 14.2(#). HR ESI-MS ($CH_3CN/CDCl_3$) *m/z* = 1149.4407 [M + Na]$^+$ (calc. for $C_{66}H_{65}N_3NaPtS$, 1149.4444). Anal. calcd. for $C_{66}H_{65}N_3PtS$: C, 70.31; H, 5.81; N, 3.73%. Found: C, 70.38; H, 6.20; N, 3.39%.

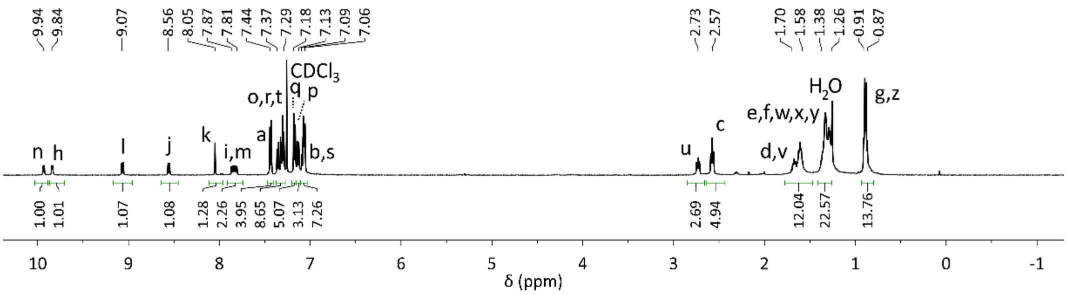

Figure S24: ^{1}H NMR spectrum (500 MHz, $CDCl_3$, 298 K) of **Pt-thio-TPA**.

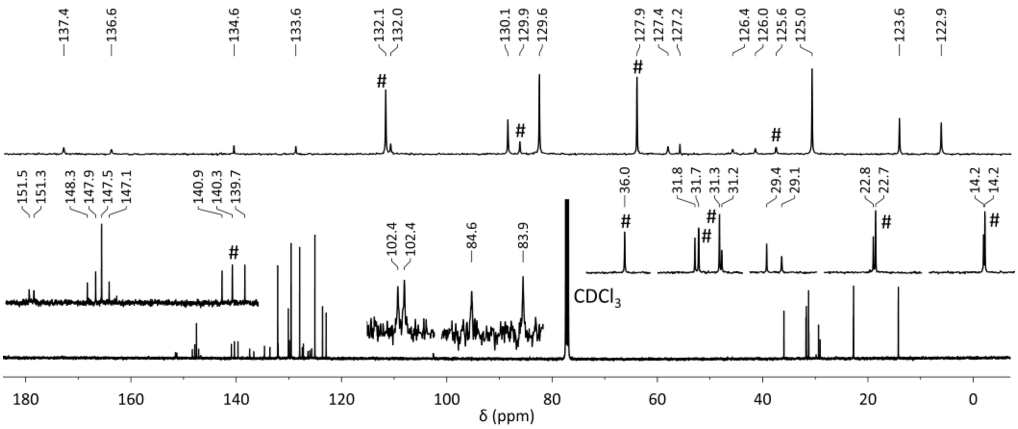

Figure S25: ^{13}C NMR spectrum (125 MHz, $CDCl_3$, 298 K) of **Pt-thio-TPA**.

Synthesis of Pt-CC-TPA

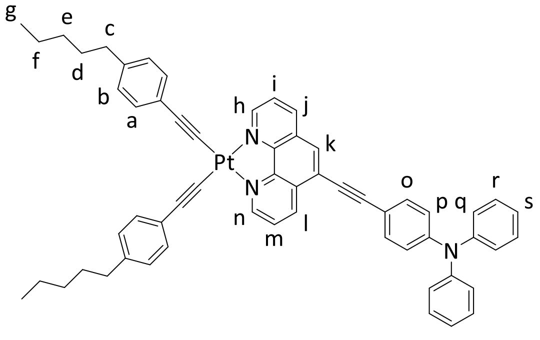

A solution of **CC-TPA** (89 mg, 0.19 mmol) and [Pt(COD)(4-pentylphenylacetylide)$_2$][3] (120 mg, 0.187 mmol) in a 1:5 CH_3CN/dichloromethanesolvent mixture (60 mL) was degassed with N_2 and heated at 45 °C under a nitrogen atmosphere for 3 days. After the removal of the solvent under vacuum, purification using column chromatography on silica (1:1 to 1:3 PE/dichloromethane, then dichloromethane, then 1:20 acetone/dichloromethane) gave the product as a red solid (140 mg, 0.138 mmol, 74%). ^{1}H NMR (500 MHz, $CDCl_3$, 298 K) *δ*: 9.88 (1H, d, *J* = 5.1 Hz, H_n), 9.82 (1H, d, *J* = 4.0 Hz, H_h), 8.85 (1H, d, *J* = 8.3 Hz, H_l), 8.51 (1H, d, *J* = 8.3 Hz, H_j), 8.06 (1H, s, H_k), 7.83 (1H, dd, *J* = 8.5, 5.1 Hz, H_m), 7.78 (1H, dd, *J* = 8.6, 5.9 Hz, H_i), 7.49 – 7.46 (6H, m, $H_{a,o}$), 7.33 (4H, d, *J* = 8.3 Hz, H_r), 7.18 – 7.11 (6H, m, $H_{q,s}$), 7.07 – 7.04 (6H, m, $H_{b,p}$), 2.60 – 2.54 (4H, m, H_c), 1.69 – 1.57 (4H, m, H_d), 1.35 – 1.30 (8H, m, $H_{e,f}$), 0.92 – 0.88 (6H, m, H_g). ^{13}C NMR (125 MHz, $CDCl_3$, 298 K, # = two coincident peaks) *δ*: 151.5, 151.2, 149.4, 147.4, 146.9, 146.8, 140.3 (#), 140.3, 137.4, 136.3, 133.2, 132.1 (#), 130.1, 130.0, 130.0, 129.7, 128.0 (#), 126.5, 126.2, 125.6 (#), 124.4, 121.9, 121.6, 113.7, 102.5, 102.4, 99.1, 84.0, 83.6, 83.5, 36.0 (#), 31.7 (#), 31.3 (#), 22.7 (#), 14.2 (#). HR ESI-MS ($CH_3CN/CDCl_3$) *m/z* = 1007.3714 [M + Na]$^+$ (calc. for $C_{58}H_{51}N_3Pt$, 1007.3628). Anal. calcd. for $C_{58}H_{51}N_3Pt{\cdot}1.2H_2O$: C, 69.20; H, 5.35; N, 4.17%. Found: 69.03; H, 5.45; N, 4.09%.

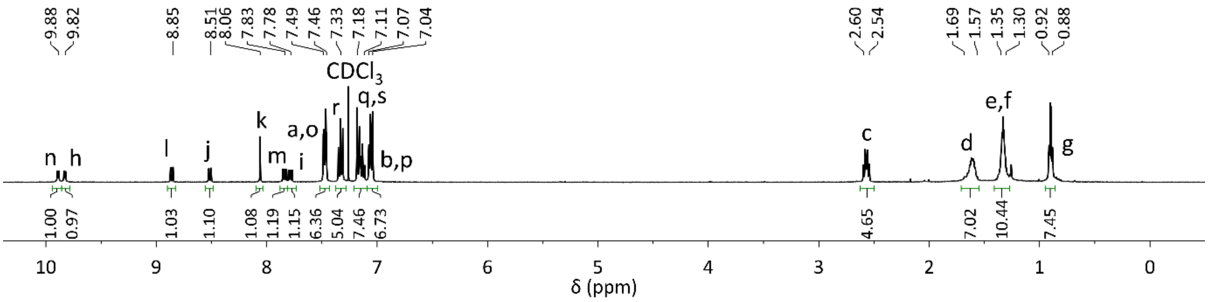

Figure S26: ^{1}H NMR spectrum (500 MHz, $CDCl_3$, 298 K) of **Pt-CC-TPA**.

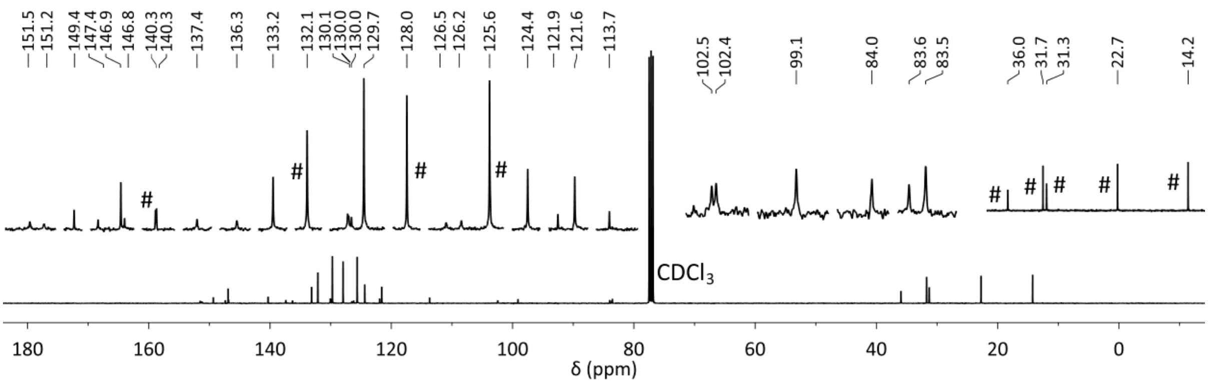

Figure S27: ^{13}C NMR spectrum (125 MHz, $CDCl_3$, 298 K) of **Pt-CC-TPA**.

Spectroscopy

All solution phase measurements were conducted in HPLC grade solvents.

Steady state spectroscopy

Electronic absorption spectra were obtained using an Ocean Optics USB2000+ UV-vis spectrophotometer with a 1 cm path length.

FT-Raman spectra were collected on solid samples in KBr disks using 1064 nm excitation provided by a Nd:YAG laser with a power between 150-500 mW. Spectra were obtained using a Bruker Optics MultiRAM spectrometer with a liquid nitrogen cooled D418T germanium detector. Spectra were collected with 4 cm^{-1} resolution, with 500-2000 accumulations depending on the sample.

Resonance Raman spectra were collected at a wide range of excitation wavelengths which were provided by both ion and diode lasers. A continuous wave Innova I-302 (Coherent) krypton ion laser was used to generate excitation wavelengths of 350.7, 406.7 and 413.2 nm and were filtered through a Pellin-Broca prism. Two irises were used to narrow the beam and reduce the power. Cobalt and CrystalLaser diode lasers were used to generate excitation wavelengths of 457 and 491 nm, and 375 and 448 nm, respectively. The beam was focused on a spinning NMR tube containing the sample (~10^{-3} M) at an approximate 135° backscattering geometry. Two planoconvex lenses were used to collimate and focus the scattered photons onto the opening slit (50 µm) of a SpectraPro 500i (Acton Research) spectrograph with a 1200 groove mm^{-1} diffraction grating. This was connected to a liquid nitrogen cooled PyLoN CCDD controlled by WinSpec/32 software. Notch filters were used to block the Rayleigh line. The spectra were calibrated at each excitation wavelength using a 1:1 mixture solution of toluene and acetonitrile.

Transient spectroscopy

Emission lifetimes of the **thio-TPA** ligand were measured by photon counting where a EPLED 320 nm ps pulsed LED (Edinburgh Instruments) was used to excite the sample. The system was run using Fluoracle v 1.9.4 software.

Transient absorption measurements were made using a LP920K (Edinburgh Instruments) system. The 3rd harmonic (354.7 nm) of a Nd:YAG pulse laser (Quantel Brilliant) operated at 10 Hz was used to excite the sample. A 450 W Xe900 exon arc lamp (Edinburgh Instruments) was used as the white-light probe source. The photons were dispersed using a TMS300-A monochrometer with a 1800 grooves mm^{-1} grating onto a R928 photomultiplier (Hamamatsu) and recorded on a TDS3012C oscilloscope (Tektronix). Transient absorption profiles were obtained using an iStar iCCD (Andor). The laser power was controlled through the use of Glan-Taylor polarisers (Thor-Labs) and measured using an Energy Max USB (Coherent) power meter. The system was controlled using L900 software (Edinburgh Instruments). Oxygen was removed from the complexes in solution via the freeze-pump-thaw method.

The time-resolve resonance Raman (TR^3) measurements were made using two Nd:YAG pulse lasers, operating at the 3rd (354.7 nm pump pulse) and 2nd (532.0 nm probe pulse) harmonic. Argon purged samples (~ 10^{-3} M in CH_2Cl_2) were placed in spinning NMR tubes and the lasers focused on the sample at an approximate 135° backscattering geometry. The time delay between the pump and probe pulses was controlled using a DG535 four channel digital delay pulse generator (Stanford Research Systems). The laser beams were overlapped using a high energy combination mirror (NewPort) and their alignment monitored using burn profiles to ensure optimal spatial overlap and a small spot size. The scattered photons were collimated and focused onto a 50 µm slit of an Acton SpectraPro 2500i spectrograph (Princeton Instruments) and dispersed by a 1200 grooves mm^{-1} grating onto a PI-MAX intensified camera (Princeton Instruments) with a Peltier cooled CCD with a ST-133 controller. 355 and 532 nm long-pass filters were used and the laser pulse power was controlled with Glan-Taylor polarisers (Thorlabs). 'Probe only' spectra were obtained when the pump pulse was blocked with a beam dump. Measurements were controlled using WinSpec/32 software.

The sample height was manually adjusted during the measurement to reduce sample degradation. Multiple spectra from freshly prepared samples were obtained at a range of time delays. The samples were argon purged prior to measurement and spectra were calibrated with a 1:1 mixture of acetonitrile and toluene. TR^3 spectra of

$[Cu(dmp)2]^+$, dmp = bis(2,9-dimethyl-1,10-phenanthroline) measured at different pump-probe delays were used as a standard to verify that the set-up was functioning correctly.[4, 5]

Computational details

All quantum chemical calculations were performed using the Gaussian 16 program[6] (B.01) (scalar relativistic simulations were performed using Orca[7, 8] – see details below). The singlet ground state geometries of the investigated structures were obtained at the density functional level of theory (DFT). A B3LYP[9, 10] based functional denoted B3LYP35[11-16] comprising of 35% exact-exchange, 58.5% of non-local B88[17] exchange and the LYP[10] correlation was employed for the complexes while the CAM-B3LYP[18] functional was used for the organic ligand structures. Unless stated otherwise, the split-valence def2-SVP basis set[19] was used, alongside Grimme's D3 dispersion correction[20] with Beck-Johnson damping to account for long-range interactions. An implicit CH_2Cl_2 solvent field was incorporated with the integral equation formalism, employing the SMD model.[21] For comparison purposes, a TDDFT calculation was also performed on **Re-thio-TPA** (rotamer A) and **Pt-thio-TPA** (rotamer A) using the triple zeta basis set def2-TZVP.

The C_6H_{13} alkyl chain of the thiophene moiety and the C_5H_{11} chain of the Pt(II) acetylide coligands shown in Figure 1, were substituted for CH_3 groups in the simulations to reduce computational time.

A relaxed scan was performed on the ligands and complexes containing the thiophene and triazole groups, where the dihedral angle shown in Figure S2 was varied in °5 increments. The DFT optimised geometry was utilised as the initial structure. The potential energy surface (PES) over this coordinate revealed the presence of four energetic minima, which were verified with frequency calculations. Time-dependent DFT (TDDFT) calculations were performed using the same respective functionals, at geometries along this coordinate and the energies of the lowest lying ^{1}ILCT and ^{1}MLCT states were obtained. The same procedure was used to obtain the T_1 rotamers in the triplet manifold. The optimised S_0 and T_1 geometries of the structures, including the various rotamers are available in Ref. [22] via the open data repository Zenodo.

Unless stated otherwise, the simulated electronic (TDDFT) and vibrational (DFT) spectra presented were generated from each of the identified rotamer structures and weighted according to their respective energies. The presented spectra thereby give a Boltzmann weighted distribution of the rotamers. Vibrational spectra were scaled by a factor of 0.95 to account for anharmonicity and insufficient treatment of electron correlation.[23]

Resonance Raman spectra were simulated within the independent mode displaced harmonic oscillator model and utilised gradients from excited states S_1-S_6. Details of the resonance Raman simulations have been reported previously.[24, 25]

Transient absorption spectra were simulated by subtracting the electronic absorption spectrum of the ground state (obtained from Boltzmann weighted spectra of the S_0 rotamers A-D), from the electronic absorption spectrum of the excited state. The excited state absorption features were obtained by spin and dipole-allowed triplet-to-triplet TDDFT calculations for the lowest 200 triplet states. The excited state spectra were scaled based on the Boltzmann weighting of the respective S_0 rotamers. In other words, based on the PESs given in Figure S3, if the S_0 rotamer A (dihedral = -45°) of **Re-thio-TPA** was excited, it would likely relax into the triplet geometry of T_1 rotamer D (dihedral = -14.6°).

Furthermore, scalar-relativistic TDDFT calculations were performed utilizing Orca 5.0.3[7] with both the Zero-Order Regular Approximation (ZORA) and the Douglas-Kroll-Hess (DKH) approach to assess prominent pathways for intersystem crossing. DFT and TDDFT calculations were performed for each rotamer of each complex in their singlet ground state geometries. The B3LYP35 functional and the ZORA-def2-SVP or DKH-def2-SVP basis sets (with the corresponding sarc-zora-svp or sarc-dkh-svp basis for the Re/Pt atoms) were applied. [26, 27] The 10 lowest singlet-singlet and singlet-triplet excitations were obtained, while spin-orbit couplings (SOCs) between these states and the singlet ground state were obtained at the SR-TDDFT level of theory using the RI-SOMF(1X) method.[7, 28] Effects of interaction with CH_2Cl_2 were taken into consideration by means of the SMD model.[21]

References

1. Pei, J.; Ni, J.; Zhou, X.-H.; Cao, X.-Y.; Lai, Y.-H., Head-to-Tail Regioregular Oligothiophene-Functionalized 9,9′-Spirobifluorene Derivatives. 1. Synthesis. *J. Org. Chem.* **2002,** *67* (14), 4924-4936.
2. Lauria, T.; Slator, C.; McKee, V.; Müller, M.; Stazzoni, S.; Crisp, A. L.; Carell, T.; Kellett, A., A Click Chemistry Approach to Developing Molecularly Targeted DNA Scissors. *Chem. Eur. J.* **2020,** *26* (70), 16782-16792.
3. Shillito, G. E.; Preston, D.; Traber, P.; Steinmetzer, J.; McAdam, C. J.; Crowley, J. D.; Wagner, P.; Kupfer, S.; Gordon, K. C., Excited-State Switching Frustrates the Tuning of Properties in Triphenylamine-Donor-Ligand Rhenium(I) and Platinum(II) Complexes. *Inorg. Chem.* **2020,** *59* (10), 6736-6746.
4. Gordon, K. C.; McGarvey, J. J., Time-resolved resonance Raman spectroscopy of bis (2, 9-dimethyl-1, 10-phenanthroline) copper (1+) in solution. *Inorg. Chem.* **1991,** *30* (15), 2986–2989.
5. McGarvey, J. J.; Bell, S. E. J.; Gordon, K. C., Single-and two-color pulsed laser resonance Raman spectroscopy of excited states of bis (2, 9-dimethyl-1, 10-phenanthroline) copper (I) in solution. *Inorg. Chem.* **1988,** *27* (22), 4003–4006.
6. Frisch, M. J.; Trucks, G. W.; Schlegel, H. B.; Scuseria, G. E.; Robb, M. A.; Cheeseman, J. R.; Scalmani, G.; Barone, V.; Petersson, G. A.; Nakatsuji, H.; Li, X.; Caricato, M.; Marenich, A. V.; Bloino, J.; Janesko, B. G.; Gomperts, R.; Mennucci, B.; Hratchian, H. P.; Ortiz, J. V.; Izmaylov, A. F.; Sonnenberg, J. L.; Williams-Young, D.; Ding, F.; Lipparini, F.; Egidi, F.; Goings, J.; Peng, B.; Petrone, A.; Henderson, T.; Ranasinghe, D.; Zakrzewski, V. G.; Gao, J.; Rega, N.; Zheng, G.; Liang, W.; Hada, M.; Ehara, M.; Toyota, K.; Fukuda, R.; Hasegawa, J.; Ishida, M.; Nakajima, T.; Honda, Y.; Kitao, O.; Nakai, H.; Vreven, T.; Throssell, K.; Montgomery, J. J. A.; Peralta, J. E.; Ogliaro, F.; Bearpark, M. J.; Heyd, J. J.; Brothers, E. N.; Kudin, K. N.; Staroverov, V. N.; Keith, T. A.; Kobayashi, R.; Normand, J.; Raghavachari, K.; Rendell, A. P.; Burant, J. C.; Iyengar, S. S.; Tomasi, J.; Cossi, M.; Millam, J. M.; Klene, M.; Adamo, C.; Cammi, R.; Ochterski, J. W.; Martin, R. L.; Morokuma, K.; Farkas, O.; Foresman, J. B.; Fox, D. J., Gaussian-16 Revision B.01. 2016.
7. Neese, F.; Wennmohs, F.; Becker, U.; Riplinger, C., The ORCA quantum chemistry program package. *J. Chem. Phys.* **2020,** *152* (22).
8. Neese, F., The ORCA program system. *WIREs Computational Molecular Science* **2012,** *2* (1), 73-78.
9. Becke, A. D., Becke's three parameter hybrid method using the LYP correlation functional. *J. Chem. Phys* **1993,** *98*, 5648-5652.
10. Lee, C.; Yang, W.; Parr, R. G., Development of the Colle-Salvetti correlation-energy formula into a functional of the electron density. *Phys. Rev. B* **1988,** *37* (2), 785-785.
11. Shillito, G. E.; Hall, T. B. J.; Preston, D.; Traber, P.; Wu, L.; Reynolds, K. E. A.; Horvath, R.; Sun, X. Z.; Lucas, N. T.; Crowley, J. D.; George, M. W.; Kupfer, S.; Gordon, K. C., Dramatic Alteration of 3ILCT Lifetimes Using Ancillary Ligands in [Re(L)(CO)3(phen-TPA)]n+ Complexes: An Integrated Spectroscopic and Theoretical Study. *J. Am. Chem. Soc.* **2018,** *140* (13), 4534-4542.
12. Kupfer, S.; Zedler, L.; Guthmuller, J.; Bode, S.; Hager, M. D.; Schubert, U. S.; Popp, J.; Gräfe, S.; Dietzek, B., Self-healing mechanism of metallopolymers investigated by QM/MM simulations and Raman spectroscopy. *Phys. Chem. Chem. Phys.* **2014,** *16* (24), 12422-12432.
13. Schindler, J.; Kupfer, S.; Zedler, L.; Wächtler, M.; Gräfe, S.; Ryan, A. A.; Senge, M. O.; Dietzek, B., Spectroelectrochemical Investigation of the One-Electron Reduction of Nonplanar Nickel (II) Porphyrins. *ChemPhysChem* **2016,** *17* (21), 3480–3493.
14. Reiher, M.; Salomon, O.; Hess, B. A., Reparameterization of hybrid functionals based on energy differences of states of different multiplicity. *Theor. Chem. Acc.* **2001,** *107* (1), 48-55.
15. Salomon, O.; Reiher, M.; Hess, B. A., Assertion and validation of the performance of the B3LYP⋆ functional for the first transition metal row and the G2 test set. *J. Chem. Phys.* **2002,** *117* (10), 4729-4737.
16. Kupfer, S.; Wächtler, M.; Guthmuller, J., Light-Driven Multi-Charge Separation in a Push-Pull Ruthenium-Based Photosensitizer – Assessed by RASSCF and TDDFT Simulations. *ChemPhotoChem* **2022,** *6* (6), e202200010.
17. Becke, A. D., Density-functional exchange-energy approximation with correct asymptotic behavior. *Phys. Rev. A* **1988,** *38* (6), 3098-3098.
18. Yanai, T.; Tew, D. P.; Handy, N. C., A new hybrid exchange–correlation functional using the Coulomb-attenuating method (CAM-B3LYP). *Chem. Phys. Lett.* **2004,** *393* (1–3), 51-57.
19. Weigend, F.; Ahlrichs, R., Balanced basis sets of split valence, triple zeta valence and quadruple zeta valence quality for H to Rn: Design and assessment of accuracy. *PCCP* **2005,** *7* (18), 3297-3305.

20. Grimme, S.; Ehrlich, S.; Goerigk, L., Effect of the damping function in dispersion corrected density functional theory. *J. Comput. Chem.* **2011,** *32* (7), 1456-1465.
21. Marenich, A. V.; Cramer, C. J.; Truhlar, D. G., Universal Solvation Model Based on Solute Electron Density and on a Continuum Model of the Solvent Defined by the Bulk Dielectric Constant and Atomic Surface Tensions. *J. Phys. Chem. B* **2009,** *113* (18), 6378-6396.
22. Shillito, G. E., DFT optimised structures of a series of bichromophoric photosensitisers. **2023.** DOI:10.5281/zenodo.10102441
23. Merrick, J. P.; Moran, D.; Radom, L., An Evaluation of Harmonic Vibrational Frequency Scale Factors. *J. Phys. Chem. A* **2007,** *111* (45), 11683-11700.
24. Guthmuller, J., Comparison of simplified sum-over-state expressions to calculate resonance Raman intensities including Franck-Condon and Herzberg-Teller effects. *J. Chem. Phys.* **2016,** *144* (6), 064106.
25. Wächtler, M.; Guthmuller, J.; González, L.; Dietzek, B., Analysis and characterization of coordination compounds by resonance Raman spectroscopy. *Coordin Chem Rev* **2012,** *256* (15-16), 1479-1508.
26. Bühl, M.; Reimann, C.; Pantazis, D. A.; Bredow, T.; Neese, F., Geometries of Third-Row Transition-Metal Complexes from Density-Functional Theory. *J. Chem. Theory Comput.* **2008,** *4* (9), 1449-1459.
27. Pantazis, D. A.; Chen, X.-Y.; Landis, C. R.; Neese, F., All-Electron Scalar Relativistic Basis Sets for Third-Row Transition Metal Atoms. *J. Chem. Theory Comput.* **2008,** *4* (6), 908-919.
28. Neese, F., Efficient and accurate approximations to the molecular spin-orbit coupling operator and their use in molecular g-tensor calculations. *J. Chem. Phys.* **2005,** *122* (3).
